# Supplementary material for: A Machine Learning-Driven Virtual Biopsy System For Kidney Transplant Patients
Source: Nat Commun. 2024 Jan 16;15:554. doi: 10.1038/s41467-023-44595-z (PMC10791605; doi:10.1038/s41467-023-44595-z)
Supplement: Supplementary file 1 — Supplementary Information [file 41467_2023_44595_MOESM1_ESM.pdf]

# Supplementary Information


**1. SUPPLEMENTARY METHODS**

**Supplementary Method 1: Population in detail, center**
**Supplementary Method 2: Systematic review**
**Supplementary Method 3: Banff grading scheme**
**Supplementary Method 4: Predictor variables**
**Supplementary Method 5: TRIPOD (adapted to the field of machine learning) checklist**
**Supplementary Method 6: Rationale for the cutoffs used to measure the performance metrics**
**Supplementary Method 7: Imputation process and included variables**

**2. SUPPLEMENTARY TABLES**

**Supplementary Table 1: Baseline donor characteristics of the derivation cohort by center**
**Supplementary Table 2: Baseline donor characteristics of the derivation cohort by donor type**
**Supplementary Table 3: Baseline donor characteristics of the derivation cohort before and after imputation comparison**
**Supplementary Table 4: Baseline donor characteristics of the external validation cohort before and after imputation comparison**
**Supplementary Table 5: Hyperparameters tuning and results**
**Supplementary Table 6: Internal validation of the virtual biopsy system during cross-validation process**
**Supplementary Table 7: Calibration confusion matrices of the virtual biopsy system (internal validation)**
**Supplementary Table 8: Comparison of donor baseline characteristics between the derivation cohort and the external validation cohort**
**Supplementary Table 9: Baseline donor characteristics of the external validation cohort by center**
**Supplementary Table 10: Baseline donor characteristics of the external validation cohort by donor type**
**Supplementary Table 11: Calibration confusion matrices of the virtual biopsy system (external validation)**
**Supplementary Table 12: Internal validation of the virtual biopsy system in various subpopulations and clinical scenarios**
**Supplementary Table 13: KDPI as a parameter in predicting day-zero kidney biopsy results**
**Supplementary Table 14: Summary of participating centers' biopsy practices and procedures**
**Supplementary Table 15: Summary of kidney day-zero histological lesion scores (Banff grading scheme)**

**3. SUPPLEMENTARY FIGURES**

**Supplementary Figure 1: Virtual biopsy system usage in real-life situation and organ allocation**
**Supplementary Figure 2: The potential utility and impact of the virtual biopsy system**

**4. ADDITIONAL SUPPLEMENTARY MATERIALS**

**Supplementary Movie 1: Video of the online application**

**5. SUPPLEMENTARY REFERENCES**

# 1. SUPPLEMENTARY METHODS

**Supplementary Method 1: Population in detail, center**

The European centers included Necker Hospital, Paris, France (n=1026), Saint-Louis Hospital, Paris, France (n=740), Toulouse Hospital, Toulouse, France (n=432), Bicêtre Hospital, Kremlin Bicêtre, France (n=396), University Hospital, Leuven, Belgium (n=753), University Hospital, Liege, Belgium (n=111), University Hospital Centre Zagreb, Zagreb, Croatia (n=453), Hospital Clínic i Provincial de Barcelona, Barcelona, Spain (n=350), Hospital Vall d'Hebrón, Barcelona, Spain (n=353), and Bellvitge University Hospital, Barcelona, Spain (n=96). The North American centers included the Mayo Clinic, Rochester, MN, USA (n=2933), OneLegacy organ procurement organization, Los Angeles, CA, USA (n=2811), Columbia University Medical Center, New York, NY, USA (n=1332), University of British Columbia, Vancouver, BC, Canada (n=417), and University of Alberta, Edmonton, AB, Canada (n=1161). The Australian center included the Royal Adelaide Hospital, Adelaide, Australia (n=370). The Chinese center included the Sun Yat-sen University, Guangzhou, China (n=298).

**Supplementary Method 2: Systematic review**

In medicine, tissue biopsies are routinely performed to determine diagnosis and guide therapeutics and prognosis assessment. In kidney transplantation, day-zero biopsies are used as baseline status of the kidney allograft to better contextualize lesions found in subsequent allograft biopsies and guide the decision-making process. However, biopsy remains an invasive and costly procedure that mobilizes human resources, thereby delaying the transplantation procedure. We searched PubMed and MEDLINE from January 2000 to January 2022, using the terms "non-invasive", "biopsy", "predict", and "machine learning" without language restrictions. Our search found overall 164 studies from all medical fields. We removed 12 studies predicting a single disease diagnosis (e.g. cancer). 124 studies used histological images and 28 were related to omics-based diagnoses. Overall, in all medical fields, there was no published study on generating a virtual biopsy to assess the presence and severity of biopsy lesions using a combination of non-invasive parameters.

**Supplementary Method 3: Banff grading scheme**

The Banff Classification of allograft pathology was created in 1991 and is revised every two years[1]. This is an international standardization of nomenclature and criteria for the histologic diagnosis of kidney allograft rejection and chronic lesion associated with long term outcomes in kidney transplantation. The 9$^{th}$ Banff conference in 2007 then a working group in 2017, approved the application of the Banff criteria to the day-zero biopsy[2,3]. The criteria are as follows:

- arteriosclerosis (Banff "cv" score) as the extent of arterial intimal thickening in the most severely affected artery.
    - cv0—No chronic vascular changes.
    - cv1—Vascular narrowing of up to 25% luminal area by fibrointimal thickening.
    - cv2—Vascular narrowing of 26 to 50% luminal area by fibrointimal thickening.
    - cv3—Vascular narrowing of more than 50% luminal area by fibrointimal thickening
- arteriolar hyalinosis (Banff "ah" score) as the extent of periodic acid Schiff (PAS)-positive arteriolar hyaline thickening.
    - ah0—No PAS-positive hyaline arteriolar thickening.
    - ah1—Mild to moderate PAS-positive hyaline thickening in at least 1 arteriole.
    - ah2—Moderate to severe PAS-positive hyaline thickening in more than 1 arteriole.
    - ah3—Severe PAS-positive hyaline thickening in many arterioles.
- Interstitial fibrosis and tubular atrophy (Banff "IFTA" score) replaced the term of chronic allograft nephropathy (Banff "CAN" score) in the Banff '05[4]. This score is based on the severity of interstitial fibrosis (Banff "ci" score) and tubular atrophy (Banff "ct" score).
    - IFTA0—No Interstitial fibrosis and tubular atrophy. No tubular atrophy (ct0) and interstitial fibrosis in up to 5% of cortical area (ci0).
    - IFTA1—Mild Interstitial fibrosis and tubular atrophy. Tubular atrophy in up to 25% of the area of cortical tubules (ct1) and interstitial fibrosis in 6 to 25% of cortical area (ci1).
    - IFTA2—Moderate Interstitial fibrosis and tubular atrophy. Tubular atrophy involving 26 to 50% of the area of cortical tubules (ct2) and interstitial fibrosis in 26 to 50% of cortical area (ci2).
    - IFTA3—Severe Interstitial fibrosis and tubular atrophy. Tubular atrophy in >50% of the area of cortical tubules (ct3) and/or interstitial fibrosis in >50% of cortical area (ci3).
- Glomerulosclerosis is the percentage defined by the number of sclerotic glomeruli divided by the total number of glomeruli.

**Supplementary Method 4: Predictor variables**

Eleven candidate predictors of kidney day-zero histological lesions, which were examined and used at donation, were as follows:

1. Donor's age: numeric value (years)
2. Donor's sex: binary value (female or male)
3. Donor type: binary value (deceased or living donor)
4. Donation after cerebrovascular death if deceased: binary value (yes or no)
5. Donation after circulatory death (DCD) if deceased: binary value (yes or no)
6. Donor's history of hypertension: binary value (yes or no)
7. Donor's history of diabetes: binary value (yes or no)
8. Donor's hepatitis C virus status: binary value (yes or no)
9. Donor's body mass index: numeric value (kg/$m^2$)
10. Donor's serum creatinine lowest at donation: numeric value (mg/dL)
11. Donor's proteinuria status: binary value (yes when dipstick greater than or equal to 1 or urine protein to creatinine ratio (UPCR, g/g) greater than or equal to 0.5 g/g, otherwise no)

In case of multiple donor creatinine for acute kidney injury (AKI), we included the serum creatinine before AKI.

## Supplementary Method 5: TRIPOD (adapted to the field of machine learning) checklist

| Section/Topic | Checklist Item | Page |
|---|---|---|
| Title | Identify the study as developing and/or validating (i.e., testing) a multivariable machine learning model, the target population, and the outcome to be predicted. | 1 |
| Abstract | Provide a summary of objectives, study design or data sources, setting, participants, sample size, predictors/features, outcome, machine learning methods, intended use of the model, results, and conclusions. | 3 |
| Background and objectives | Explain the medical context (including whether diagnostic or prognostic) and rationale for developing or validating the multivariable machine learning model, including references to existing models, and the main advantages of the used design and analyses. | 4-5 |
| | Explain the intended purpose (e.g., for prognosis or diagnostic predictions) and use for the model in the context of the clinical pathway, including its intended users (e.g., healthcare professionals, patients, public). | 4-5 |
| | Specify the study objectives, including whether the study describes the development or validation (e.g., testing) of the model or both. | 4-5 |
| **Data collection and preparation** | | |
| Source of data | Describe the study design or source of data (e.g., randomized trial, cohort, routine care or registry data), separately for the development and validation (test) datasets, if applicable. | 16; Supplementary method 1 |
| | Specify the key dates of the collected participant data, including start and of participant/data accrual; and, if applicable, end of follow-up. | 16 |
| Participants | Specify key elements of the study setting (e.g., primary care, secondary care, general population) including the number and location of centers or data sources. | Supplementary method 1; Supplementary table 14 |
| | Describe the eligibility criteria for participants or data sources: how, where, and when potentially eligible participants were identified (e.g., symptoms, results from previous tests, inclusion in the registry, patient-care setting, location). | 16 |
| Outcome | Clearly define the outcome (e.g., ground truth or reference standard) that is predicted by the machine learning model (including the time horizon), including how and when assessed and the rationale for choosing this outcome measurement (if alternatives exist). | 17; Supplementary method 3; Supplementary table 15 |
| Predictors | Clearly define all predictors/features used in developing the multivariable machine learning model, including how and when they were measured. Consider using supplementary material for large numbers of predictors. | 18; Supplementary method 4 |
| **Machine learning model development** | | |
| Participants | Describe the flow of participants through the study, including the number of participants with and without the outcome and, if applicable, a summary of the follow-up time. A diagram may be helpful. | Table 1; Supplementary tables 1 and 9 |
| | Describe the characteristics of the participants (basic demographics, clinical features, available predictors), including the number of participants with missing data for predictors and outcome. | Table 1; Supplementary tables 1, 3, 4, 9, and 10 |
| | For external validation (testing), show a comparison with the development data of the distribution of important predictors/features (demographics, predictors, and outcome). | Supplementary table 8 |
| Data preparation and preprocessing | Describe how missing data were handled (e.g., complete-case analysis, single imputation, multiple imputation) with details of any imputation method. | 20; Supplementary method 7; Supplementary tables 3 and 4 |
| | Specify data splitting for train (derivation) and test (validation) sets creation. | 16 |
| | Describe how unbalanced data were handled, if relevant. | 18 |
| Model development | Specify the number of participants and outcome events in each analysis. | Supplementary tables 3 and 4 |
| | Specify which machine learning model(s) is (are) used. | 19 |
| | Describe how predictors were handled in the analyses. | Supplementary method 4 |
| | Specify the resampling method used on training or derivation set. | 19 |
| Model performance | Specify type of model, all model-building procedures (including any predictor selection), and method for validation. | 19-20 |

| | | |
|---|---|---|
| | Specify all measures used to assess model performance and, if relevant, to compare multiple models. | 19-20 |
| | Report discrimination performance measures (with CIs) for the prediction model. | 7-8; Table 2, Figure 2 |
| | Present calibration performance measures for the machine learning model, if relevant. | Supplementary tables 7 and 11 |
| | Present any robustness assessment and durability test used, if relevant. | 9-10; Supplementary table 12 |
| Model specification | Present the parameter importance evaluation. | 7; Figure 1 |
| | Specify the output of the model (e.g., probabilities, classification, risk grouping). | 10 |
| | Explain how to use the prediction model. | 10; Figure 3; Supplementary figures 1 and 2; Supplementary video |
| **Findings perspective** | | |
| Limitations | Discuss any limitations of the study (such as non-representative sample, few events per predictor, missing data). | 13-14 |
| Interpretation | For validation, discuss the results with reference to performance in the cross-validation data, and any other test data. | Supplementary table 6 |
| | Give an overall interpretation of the results, considering objectives, limitations, results from similar studies, and other relevant evidence. | 11-15 |
| Implications | Discuss the potential clinical use of the model and implications for future research. | 12-13 |
| Supplementary information | Provide information about the availability of supplementary resources, such as study protocol, Web calculator, and data sets. | Supplementary information |
| Funding | Give the source of funding and the role of the funders for the present study. | 28 |

**Supplementary Method 6: Rationale for the cutoffs used to measure the performance metrics**

We used Youden's J statistic to calibrate probability score cutoffs of the ensemble models. Each ensemble model was to optimize Youden's statistic, namely, the sum of sensitivity and specificity, based on 3-times repeated 10-folds cross-validations (30 resamples). On each fold, the best cutoff was calculated then 30 cutoffs were averaged. The computed cutoffs were then used for internal and external validations to measure the performance such as sensitivity and specificity.

**Supplementary Method 7: Imputation process and included variables**

We imputed the missing values with random forest machine learning algorithm, which was implemented in missForest R package[5]. The donor parameters and biopsy findings used in the imputation algorithm were i) age, ii) sex, iii) donor type (living or deceased donor), iv) cerebrovascular cause of death, v) donor after circulatory death (DCD), vi) history of hypertension, vii) diabetes, viii) hepatitis C virus (HCV) status, ix) body mass index (BMI), x), kidney function defined by serum creatinine, xi) proteinuria status, xii) arteriosclerosis (Banff cv score), xiii) arteriolar hyalinosis (Banff ah score), xiv) interstitial fibrosis and tubular atrophy (Banff IFTA score), xv) percentage of sclerotic glomeruli (glomerulosclerosis score). The maximum number of iterations was set to 10 times. Outcome variables (Banff lesions) were not imputed.

## 2. SUPPLEMENTARY TABLES

**Supplementary Table 1 | Baseline donor characteristics of the derivation cohort by center**

| | Overall (n=12,402) | Mayo Clinic (n=2,933) | OneLegacy OPO (n=2,811) | University of Alberta (n=1,161) | Necker hospital (n=1,026) |
|---|---|---|---|---|---|
| Age (years), mean (SD) | 46.7 (14.9) | 43.4 (12.5) | 43.7 (15.0) | 40.6 (13.2) | 53.2 (16.8) |
| Sex female, No. (%) | 5450 (44.0%) | 1602 (54.6%) | 979 (34.8%) | 565 (48.7%) | 434 (42.3%) |
| Donor type | | | | | |
| Deceased donor, No. (%) | 9395 (75.8%) | 591 (20.2%) | 2811 (100.0%) | 765 (65.9%) | 1026 (100.0%) |
| Death from circulatory disease, No. (%)* | 1471 (15.7%) | 0 (0.0%) | 531 (18.9%) | 25 (3.3%) | 25 (2.4%) |
| Death from cerebrovascular disease, No. (%)* | 4001 (42.8%) | 0 (0.0%) | 942 (33.5%) | 254 (33.2%) | 583 (56.8%) |
| Diabetes mellitus, No. (%) | 782 (7.4%) | 0 (0.0%) | 428 (15.8%) | 30 (3.2%) | 76 (7.7%) |
| Hypertension, No. (%) | 2375 (21.1%) | 0 (0.0%) | 916 (33.7%) | 103 (11.2%) | 257 (26.1%) |
| BMI (kg/$m^2$), mean (SD) | 26.9 (5.5) | 27.7 (5.0) | 28.5 (6.7) | 26.1 (5.3) | 25.0 (4.7) |
| HCV status, No. (%) | 233 (1.9%) | 4 (0.1%) | 176 (6.3%) | 7 (0.7%) | 24 (2.3%) |
| Creatinine (mg/dL), mean (SD) | 1.2 (1.0) | 1.0 (0.4) | 2.0 (1.6) | 0.9 (0.6) | 1.0 (0.7) |
| Proteinuria, No. (%) | 1904 (20.7%) | 0 (0.0%) | 317 (11.4%) | 122 (33.8%) | 558 (56.5%) |
| Number of Glomeruli, mean (SD) | 39.3 (33.5) | 21.2 (10.9) | 83.9 (34.9) | 35.2 (27.7) | 18.6 (7.8) |
| Arteriosclerosis (cv) Banff score, No. (%) | | | | | |
| 0 | 7073 (60.2%) | 1714 (60.6%) | 1983 (70.5%) | 626 (66.2%) | 302 (30.6%) |
| 1 | 3105 (26.4%) | 931 (32.9%) | 404 (14.4%) | 240 (25.4%) | 328 (33.2%) |
| 2 | 1325 (11.3%) | 181 (6.4%) | 301 (10.7%) | 78 (8.2%) | 321 (32.5%) |
| 3 | 252 (2.1%) | 2 (0.1%) | 123 (4.4%) | 2 (0.2%) | 36 (3.6%) |
| Arteriolar hyalinosis (ah) Banff score, No. (%) | | | | | |
| 0 | 8242 (68.8%) | 2341 (84.0%) | 2516 (89.5%) | 624 (55.9%) | 323 (31.8%) |
| 1 | 2546 (21.3%) | 391 (14.0%) | 157 (5.6%) | 334 (29.9%) | 398 (39.1%) |
| 2 | 968 (8.1%) | 52 (1.9%) | 89 (3.2%) | 145 (13.0%) | 248 (24.4%) |
| 3 | 217 (1.8%) | 3 (0.1%) | 49 (1.7%) | 14 (1.3%) | 48 (4.7%) |
| Interstitial fibrosis and tubular atrophy (IFTA) Banff score, No. (%) | | | | | |
| 0 | 7822 (64.4%) | 2269 (79.6%) | 1803 (64.1%) | 571 (56.5%) | 660 (65.4%) |
| 1 | 3647 (30.0%) | 576 (20.2%) | 653 (23.2%) | 395 (39.1%) | 297 (29.4%) |
| 2 | 562 (4.6%) | 5 (0.2%) | 288 (10.2%) | 44 (4.4%) | 43 (4.3%) |
| 3 | 117 (1.0%) | 1 (<0.1%) | 67 (2.4%) | 1 (0.1%) | 9 (0.9%) |
| Glomerulosclerosis, median (interquartile range) | 3.0 (0.0-10.0) | 2.4 (0.0-8.3) | 0.0 (0.0-6.0) | N/A | 5.6 (0.0-12.5) |

**Supplementary Table 1 | Baseline donor characteristics of the population cohort by center (continued)**

| | KU LEUVEN (n=753) | Saint Louis hospital (n=740) | University Hospital Centre Zagreb (n=453) | Centre Hospitalier Universitaire de Toulouse (n=432) | University of British Columbia (n=417) |
|---|---|---|---|---|---|
| Age (years), mean (SD) | 46.4 (13.0) | 49.1 (15.3) | 47.8 (12.2) | 49.2 (14.3) | 45.8 (13.6) |
| Sex female, No. (%) | 337 (44.8%) | 284 (38.4%) | 186 (41.1%) | 209 (48.4%) | 192 (46.0%) |
| Donor type | | | | | |
| Deceased donor, No. (%) | 753 (100.0%) | 740 (100.0%) | 453 (100.0%) | 366 (84.7%) | 300 (71.9%) |
| Death from circulatory disease, No. (%)* | 177 (23.5%) | 71 (9.6%) | 0 (0.0%) | 0 (0.0%) | 101 (33.7%) |
| Death from cerebrovascular disease, No. (%)* | 375 (49.8%) | 429 (58%) | 283 (62.5%) | 197 (53.8%) | 72 (24%) |
| Diabetes mellitus, No. (%) | N/A | 32 (4.4%) | 7 (1.5%) | 20 (4.8%) | 18 (4.6%) |
| Hypertension, No. (%) | 98 (13.0%) | 155 (21.2%) | 125 (27.6%) | 61 (17.3%) | 42 (10.9%) |
| BMI (kg/$m^2$), mean (SD) | 25.2 (4.1) | 25.0 (4.6) | 26.3 (3.6) | 25.4 (4.2) | 27.1 (5.3) |
| HCV status, No. (%) | 0 (0.0%) | 2 (0.3%) | 0 (0.0%) | 6 (2.0%) | 9 (2.2%) |
| Creatinine (mg/dL), mean (SD) | 0.8 (0.5) | 1.0 (0.5) | 0.9 (0.4) | 1.0 (0.5) | 1.0 (0.8) |
| Proteinuria, No. (%) | N/A | 265 (39.5%) | 92 (20.3%) | 109 (41.4%) | 144 (37.2%) |
| Number of Glomeruli, mean (SD) | N/A | 19.1 (7.4) | 57.0 (33.9) | 16.6 (5.1) | 26.6 (12.0) |
| Arteriosclerosis (cv) Banff score, No. (%) | | | | | |
| 0 | 664 (88.2%) | 252 (35.5%) | 303 (66.9%) | 222 (54.5%) | 381 (91.4%) |
| 1 | 70 (9.3%) | 245 (34.5%) | 140 (30.9%) | 112 (27.5%) | 23 (5.5%) |
| 2 | 19 (2.5%) | 186 (26.2%) | 6 (1.3%) | 54 (13.3%) | 11 (2.6%) |
| 3 | 0 (0.0%) | 27 (3.8%) | 4 (0.9%) | 19 (4.7%) | 2 (0.5%) |
| Arteriolar hyalinosis (ah) Banff score, No. (%) | | | | | |
| 0 | 546 (72.5%) | 284 (38.7%) | 249 (55.0%) | 211 (50.1%) | 207 (49.6%) |
| 1 | 169 (22.4%) | 310 (42.2%) | 178 (39.3%) | 139 (33.0%) | 81 (19.4%) |
| 2 | 35 (4.6%) | 105 (14.3%) | 23 (5.1%) | 60 (14.3%) | 106 (25.4%) |
| 3 | 3 (0.4%) | 35 (4.8%) | 3 (0.7%) | 11 (2.6%) | 23 (5.5%) |
| Interstitial fibrosis and tubular atrophy (IFTA) Banff score, No. (%) | | | | | |
| 0 | 545 (72.4%) | 480 (65.0%) | 165 (36.4%) | 327 (75.7%) | 259 (62.1%) |
| 1 | 191 (25.4%) | 204 (27.6%) | 256 (56.5%) | 99 (22.9%) | 154 (36.9%) |
| 2 | 13 (1.7%) | 41 (5.5%) | 31 (6.8%) | 4 (0.9%) | 4 (1.0%) |
| 3 | 4 (0.5%) | 14 (1.9%) | 1 (0.2%) | 2 (0.5%) | 0 (0.0%) |
| Glomerulosclerosis, median (interquartile range) | 0.0 (0.0-8.3) | 5.3 (0.0-12.5) | 3.3 (0.0-7.7) | 6.2 (0.0-14.3) | 4.8 (0.0-9.5) |

**Supplementary Table 1 | Baseline donor characteristics of the population cohort by center (continued)**

| | Bicêtre hospital (n=396) | Royal Adelaide Hospital (n=370) | Vall d'Hebron University Hospital (n=353) | Hospital Clinic i Provincial de Barcelona (n=350) | Centre hospitalier universitaire de Liège (n=111) | Hospital Universitari de Bellvitge (n=96) |
|---|---|---|---|---|---|---|
| Age (years), mean (SD) | 57.2 (16.4) | 46.9 (14.4) | 61.3 (11.7) | 60.3 (10.2) | 44.4 (11.4) | 63.2 (12.1) |
| Sex female, No. (%) | 145 (36.6%) | 187 (51.9%) | 142 (40.2%) | 102 (29.1%) | 49 (44.1%) | 37 (38.5%) |
| Donor type | | | | | | |
| Deceased donor, No. (%) | 396 (100.0%) | 284 (76.8%) | 353 (100.0%) | 350 (100.0%) | 111 (100.0%) | 96 (100.0%) |
| Death from circulatory disease, No. (%)* | 99 (25%) | 65 (23%) | 103 (29.2%) | 187 (53.4%) | 48 (43.2%) | 39 (40.6%) |
| Death from cerebrovascular disease, No. (%)* | 182 (46%) | 113 (40.5%) | 207 (58.6%) | 269 (76.9%) | 58 (52.3%) | 37 (38.5%) |
| Diabetes mellitus, No. (%) | 47 (12.0%) | 10 (2.7%) | 61 (17.3%) | 35 (10.0%) | 3 (3.3%) | 15 (19.2%) |
| Hypertension, No. (%) | 140 (35.7%) | 47 (12.8%) | 198 (56.2%) | 171 (48.9%) | 24 (25.3%) | 38 (48.7%) |
| BMI (kg/$m^2$), mean (SD) | 26.0 (5.1) | 26.8 (5.7) | 27.8 (5.8) | 27.5 (4.2) | 25.9 (4.3) | 27.7 (4.9) |
| HCV status, No. (%) | 2 (0.5%) | 0 (0.0%) | 0 (0.0%) | 3 (0.9%) | 0 (0.0%) | 0 (0.0%) |
| Creatinine (mg/dL), mean (SD) | 1.0 (0.5) | 0.8 (0.3) | 0.9 (0.4) | 1.2 (0.6) | 0.8 (0.4) | 0.9 (0.3) |
| Proteinuria, No. (%) | 169 (53.1%) | 3 (3.7%) | 38 (13.1%) | 36 (23.4%) | 42 (42.4%) | 9 (45.0%) |
| Number of Glomeruli, mean (SD) | 39.2 (30.9) | 38.2 (17.4) | N/A | N/A | 27.6 (16.7) | N/A |
| Arteriosclerosis (cv) Banff score, No. (%) | | | | | | |
| 0 | 139 (36.9%) | 81 (51.3%) | 133 (37.7%) | 125 (35.8%) | 101 (91.0%) | 47 (49.5%) |
| 1 | 133 (35.3%) | 48 (30.4%) | 184 (52.1%) | 210 (60.2%) | 6 (5.4%) | 31 (32.6%) |
| 2 | 84 (22.3%) | 18 (11.4%) | 35 (9.9%) | 13 (3.7%) | 3 (2.7%) | 15 (15.8%) |
| 3 | 21 (5.6%) | 11 (7.0%) | 1 (0.3%) | 1 (0.3%) | 1 (0.9%) | 2 (2.1%) |
| Arteriolar hyalinosis (ah) Banff score, No. (%) | | | | | | |
| 0 | 192 (52.3%) | 280 (79.3%) | 177 (50.4%) | 135 (72.6%) | 94 (85.5%) | 63 (65.6%) |
| 1 | 103 (28.1%) | 63 (17.8%) | 136 (38.7%) | 51 (27.4%) | 9 (8.2%) | 27 (28.1%) |
| 2 | 49 (13.4%) | 8 (2.3%) | 36 (10.3%) | 0 (0.0%) | 6 (5.5%) | 6 (6.2%) |
| 3 | 23 (6.3%) | 2 (0.6%) | 2 (0.6%) | 0 (0.0%) | 1 (0.9%) | 0 (0.0%) |
| Interstitial fibrosis and tubular atrophy (IFTA) Banff score, No. (%) | | | | | | |
| 0 | 127 (32.4%) | 328 (88.6%) | 40 (11.3%) | 130 (37.1%) | 103 (92.8%) | 15 (15.6%) |
| 1 | 206 (52.6%) | 37 (10.0%) | 289 (81.9%) | 214 (61.1%) | 7 (6.3%) | 69 (71.9%) |
| 2 | 43 (11.0%) | 4 (1.1%) | 24 (6.8%) | 6 (1.7%) | 1 (0.9%) | 11 (11.5%) |
| 3 | 16 (4.1%) | 1 (0.3%) | 0 (0.0%) | 0 (0.0%) | 0 (0.0%) | 1 (1.0%) |
| Glomerulosclerosis, median (interquartile range) | 8.3 (2.0-15.5) | 3.9 (0.0-9.1) | 5.1 (2.0-10.0) | 7.4 (7.4-7.4)† | 0.0 (0.0-6.5) | 7.4 (0.0-7.4)† |

**Supplementary Table 2 | Baseline donor characteristics of the derivation cohort by donor type**

| | N | Overall (n=12,402) | N | Deceased Donor (n=9,395) | N | Living Donor (n=3,007) | p-value |
|---|---|---|---|---|---|---|---|
| Age (years), mean (SD) | 12402 | 46.7 (14.9) | 9395 | 47.3 (15.7) | 3007 | 44.8 (12.0) | <0.001 |
| Sex female, No. (%) | 12391 | 5450 (44.0%) | 9389 | 3659 (39.0%) | 3002 | 1791 (59.7%) | <0.001 |
| Donor type | | | | | | | |
| Deceased donor, No. (%) | 12402 | 9395 (75.8%) | 9395 | 9395 (100.0%) | 3007 | 0 (0.0%) | <0.001 |
| Death from circulatory disease, No. (%)* | 9360 | 1471 (15.7%) | 9360 | 1471 (15.7%) | 0 | 0 (-) | <0.001 |
| Death from cerebrovascular disease, No. (%)* | 9354 | 4001 (42.8%) | 9354 | 4001 (42.8%) | 0 | 0 (-) | <0.001 |
| Diabetes mellitus, No. (%) | 10585 | 782 (7.4%) | 7791 | 779 (10.0%) | 2794 | 3 (0.1%) | <0.001 |
| Hypertension, No. (%) | 11274 | 2375 (21.1%) | 8480 | 2360 (27.8%) | 2794 | 15 (0.5%) | <0.001 |
| BMI (kg/$m^2$), mean (SD) | 11456 | 26.9 (5.5) | 8538 | 26.7 (5.7) | 2918 | 27.4 (4.9) | <0.001 |
| HCV status, No. (%) | 12004 | 233 (1.9%) | 9104 | 233 (2.6%) | 2900 | 0 (0.0%) | <0.001 |
| Creatinine (mg/dL), mean (SD) | 10924 | 1.2 (1.0) | 8583 | 1.3 (1.1) | 2341 | 0.9 (0.2) | <0.001 |
| Proteinuria, No. (%) | 9218 | 1904 (20.7%) | 6609 | 1904 (28.8%) | 2609 | 0 (0.0%) | <0.001 |
| Number of Glomeruli, mean (SD) | 6993 | 39.3 (33.5) | 5903 | 41.5 (35.3) | 1090 | 27.2 (17.0) | <0.001 |
| Arteriosclerosis (cv) Banff score, No. (%) | 11755 | | 8975 | | 2780 | | <0.001 |
| 0 | | 7073 (60.2%) | | 5334 (59.4%) | | 1739 (62.6%) | |
| 1 | | 3105 (26.4%) | | 2228 (24.8%) | | 877 (31.5%) | |
| 2 | | 1325 (11.3%) | | 1163 (13.0%) | | 162 (5.8%) | |
| 3 | | 252 (2.1%) | | 250 (2.8%) | | 2 (0.1%) | |
| Arteriolar hyalinosis (ah) Banff score, No. (%) | 11973 | | 9108 | | 2865 | | <0.001 |
| 0 | | 8242 (68.8%) | | 5918 (65.0%) | | 2324 (81.1%) | |
| 1 | | 2546 (21.3%) | | 2097 (23.0%) | | 449 (15.7%) | |
| 2 | | 968 (8.1%) | | 878 (9.6%) | | 90 (3.1%) | |
| 3 | | 217 (1.8%) | | 215 (2.4%) | | 2 (0.1%) | |
| Interstitial fibrosis and tubular atrophy (IFTA) Banff score, No. (%) | 12148 | | 9285 | | 2863 | | <0.001 |
| 0 | | 7822 (64.4%) | | 5606 (60.4%) | | 2216 (77.4%) | |
| 1 | | 3647 (30.0%) | | 3014 (32.5%) | | 633 (22.1%) | |
| 2 | | 562 (4.6%) | | 549 (5.9%) | | 13 (0.5%) | |
| 3 | | 117 (1.0%) | | 116 (1.2%) | | 1 (<0.1%) | |
| Glomerulosclerosis, median (interquartile range) | 8826 | 3.0 (0.0-10.0) | 8151 | 3.0 (0.0-10.0) | 675 | 4.2 (0.0-9.1) | 0.934 |

**Supplementary Table 3 | Baseline donor characteristics of the derivation cohort before and after imputation comparison**

| | N | Before Imputation (n=12,402) | N | After Imputation (n=12,402) | p-value |
|---|---|---|---|---|---|
| Age (years), mean (SD) | 12402 | 46.7 (14.9) | 12402 | 46.7 (14.9) | 1.00 |
| Sex female, No. (%) | 12391 | 5450 (44.0%) | 12402 | 5454 (44.0%) | 1.00 |
| Donor type, No. (%) | | | | | |
| Deceased donor, No. (%) | 12402 | 9395 (75.8%) | 12402 | 9395 (75.8%) | 1.00 |
| Death from circulatory disease, No. (%)* | 9360 | 1471 (15.7%) | 9395 | 1475 (15.7%) | 0.992 |
| Death from cerebrovascular disease, No. (%)* | 9354 | 4001 (42.8%) | 9395 | 4009 (42.7%) | 0.900 |
| Diabetes mellitus, No. (%) | 10585 | 782 (7.4%) | 12402 | 809 (6.5%) | 0.0108 |
| Hypertension, No. (%) | 11274 | 2375 (21.1%) | 12402 | 2497 (20.1%) | 0.0791 |
| BMI (kg/$m^2$), mean (SD) | 11456 | 26.9 (5.5) | 12402 | 26.8 (5.3) | 0.339 |
| HCV status, No. (%) | 12004 | 233 (1.9%) | 12402 | 243 (2.0%) | 0.954 |
| Creatinine (mg/dL), mean (SD) | 10924 | 1.2 (1.0) | 12402 | 1.2 (1.0) | 0.325 |
| Proteinuria, No. (%) | 9218 | 1904 (20.7%) | 12402 | 3307 (26.7%) | <0.001 |
| Number of Glomeruli, mean (SD) | 6993 | 39.3 (33.5) | 6993 | 39.3 (33.5) | 1.00 |
| Arteriosclerosis (cv) Banff score, No. (%) | 11755 | | 11755 | | 1.00 |
| 0 | | 7073 (60.2%) | | 7073 (60.2%) | |
| 1 | | 3105 (26.4%) | | 3105 (26.4%) | |
| 2 | | 1325 (11.3%) | | 1325 (11.3%) | |
| 3 | | 252 (2.1%) | | 252 (2.1%) | |
| Arteriolar hyalinosis (ah) Banff score, No. (%) | 11973 | | 11973 | | 1.00 |
| 0 | | 8242 (68.8%) | | 8242 (68.8%) | |
| 1 | | 2546 (21.3%) | | 2546 (21.3%) | |
| 2 | | 968 (8.1%) | | 968 (8.1%) | |
| 3 | | 217 (1.8%) | | 217 (1.8%) | |
| Interstitial fibrosis and tubular atrophy (IFTA) Banff score, No. (%) | 12148 | | 12148 | | 1.00 |
| 0 | | 7822 (64.4%) | | 7822 (64.4%) | |
| 1 | | 3647 (30.0%) | | 3647 (30.0%) | |
| 2 | | 562 (4.6%) | | 562 (4.6%) | |
| 3 | | 117 (1.0%) | | 117 (1.0%) | |
| Glomerulosclerosis, median (interquartile range) | 8826 | 3.0 (0.0-10.0) | 8826 | 3.0 (0.0-10.0) | 1.00 |

**Supplementary Table 4 | Baseline donor characteristics of the external validation cohort before and after imputation comparison**

| | N | Before Imputation (n=1,630) | N | After Imputation (n=1,630) | p-value |
|---|---|---|---|---|---|
| Age (years), mean (SD) | 1630 | 48.0 (13.2) | 1630 | 48.0 (13.2) | 1.00 |
| Sex female, No. (%) | 1630 | 723 (44.4%) | 1630 | 723 (44.4%) | 1.00 |
| Donor type, No. (%) | | | | | |
| Deceased donor, No. (%) | 1630 | 1124 (69.0%) | 1630 | 1124 (69.0%) | 1.00 |
| Death from circulatory disease, No. (%)* | 1124 | 131 (11.7%) | 1630 | 131 (11.7%) | 1.00 |
| Death from cerebrovascular disease, No. (%)* | 1124 | 525 (46.7%) | 1630 | 525 (46.7%) | 1.00 |
| Diabetes mellitus, No. (%) | 1615 | 215 (13.3%) | 1630 | 223 (13.7%) | 0.798 |
| Hypertension, No. (%) | 1618 | 594 (36.7%) | 1630 | 605 (37.1%) | 0.839 |
| BMI (kg/$m^2$), mean (SD) | 1624 | 27.6 (6.3) | 1630 | 27.6 (6.3) | 0.979 |
| HCV status, No. (%) | 1630 | 49 (3.0%) | 1630 | 49 (3.0%) | 1.00 |
| Creatinine (mg/dL), mean (SD) | 1217 | 1.6 (1.1) | 1630 | 1.5 (1.0) | <0.001 |
| Proteinuria, No. (%) | 1623 | 587 (36.2%) | 1630 | 593 (36.4%) | 0.928 |
| Number of Glomeruli, mean (SD) | 971 | 54.7 (45.0) | 971 | 54.7 (45.0) | 1.00 |
| Arteriosclerosis (cv) Banff score, No. (%) | 1625 | | 1625 | | 1.00 |
| 0 | | 453 (27.9%) | | 453 (27.9%) | |
| 1 | | 551 (33.9%) | | 551 (33.9%) | |
| 2 | | 590 (36.3%) | | 590 (36.3%) | |
| 3 | | 31 (1.9%) | | 31 (1.9%) | |
| Arteriolar hyalinosis (ah) Banff score, No. (%) | 953 | | 953 | | 1.00 |
| 0 | | 513 (53.8%) | | 513 (53.8%) | |
| 1 | | 366 (38.4%) | | 366 (38.4%) | |
| 2 | | 61 (6.4%) | | 61 (6.4%) | |
| 3 | | 13 (1.4%) | | 13 (1.4%) | |
| Interstitial fibrosis and tubular atrophy (IFTA) Banff score, No. (%) | 1630 | | 1630 | | 1.00 |
| 0 | | 658 (40.4%) | | 658 (40.4%) | |
| 1 | | 501 (30.7%) | | 501 (30.7%) | |
| 2 | | 467 (28.7%) | | 467 (28.7%) | |
| 3 | | 4 (0.2%) | | 4 (0.2%) | |
| Glomerulosclerosis, median (interquartile range) | 1629 | 2.1 (0.0-12.5) | 1629 | 2.1 (0.0-12.5) | 1.00 |

**Supplementary Table 5 | Hyperparameters tuning and results**

**Supplementary Table 5.1 | Arteriosclerosis (Banff cv score)**

| Machine learning models | Hyperparameters |
|---|---|
| Random Forest | mtry=4 |
| Gradient Boosting Machine | n.trees=700<br>interaction.depth=13<br>shrinkage=0.01<br>n.minobsinnode=7 |
| Extreme Gradient Boosting Tree | nrounds=54<br>max_depth=18<br>eta=0.1852479<br>gamma=0.02767602<br>colsample_bytree=0.6063756<br>min_child_weight=0.9<br>subsample=0.790576 |
| Linear Discriminant Analysis | - |
| Model Averaged Neural Network | size=25<br>decay=0.1<br>bag=TRUE |
| Multinomial Logistic Regression | decay=0.001 |

**Supplementary Table 5.2 | Arteriolar hyalinosis (Banff ah score)**

| Machine learning models | Hyperparameters |
|---|---|
| Random Forest | mtry=4 |
| Gradient Boosting Machine | n.trees=700<br>interaction.depth=13<br>shrinkage=0.01<br>n.minobsinnode=5 |
| Extreme Gradient Boosting Tree | nrounds=27<br>max_depth=18<br>eta=0.06210775<br>gamma=0.01385926<br>colsample_bytree=0.8300242<br>min_child_weight=1.1<br>subsample=0.8261786 |
| Linear Discriminant Analysis | - |
| Model Averaged Neural Network | size=15<br>decay=0.1<br>bag=TRUE |
| Multinomial Logistic Regression | decay=0.001 |

**Supplementary Table 5.3 | Interstitial fibrosis and tubular atrophy (Banff IFTA score)**

| Machine learning models | Hyperparameters |
|---|---|
| Random Forest | mtry=4 |
| Gradient Boosting Machine | n.trees=700<br>interaction.depth=13<br>shrinkage=0.01<br>n.minobsinnode=7 |
| Extreme Gradient Boosting Tree | nrounds=38<br>max_depth=15<br>eta=0.1508891<br>gamma=0.04430697<br>colsample_bytree=0.5812269<br>min_child_weight=1.9<br>subsample=0.9993576 |
| Linear Discriminant Analysis | - |
| Model Averaged Neural Network | size=10<br>decay=0.01<br>bag=FALSE |
| Multinomial Logistic Regression | decay=0.01 |

**Supplementary Table 5.4 | Glomerulosclerosis (percentage of sclerotic glomeruli)**

| Machine learning models | Hyperparameters |
|---|---|
| Random Forest | mtry=8 |
| Gradient Boosting Machine | n.trees=700<br>interaction.depth=13<br>shrinkage=0.01<br>n.minobsinnode=5 |
| Extreme Gradient Boosting Tree | nrounds=283<br>max_depth=18<br>eta=0.01032906<br>gamma=0.04123139<br>colsample_bytree=0.5279746<br>min_child_weight=0.7<br>subsample=0.6965341 |
| Model Averaged Neural Network | size=10<br>decay=0.01<br>bag=FALSE |
| Ensemble model (linear regression) | - |

**Supplementary Table 6 | Internal validation of the virtual biopsy system during cross-validation process**

**Supplementary Table 6.1 | Internal validation of the virtual biopsy system during cross-validation process (cv score)**

| Resample | Random Forest | Gradient Boosting Machine | Extreme Gradient Boosting Tree | Linear Discriminant Analysis | Model Averaged Neural Network | Multinomial logistic regression | Ensemble model |
|---|---|---|---|---|---|---|---|
| Fold01.Rep1 | 0.844 | 0.806 | 0.844 | 0.741 | 0.760 | 0.735 | 0.826 |
| Fold02.Rep1 | 0.834 | 0.819 | 0.839 | 0.741 | 0.766 | 0.740 | 0.827 |
| Fold03.Rep1 | 0.834 | 0.785 | 0.806 | 0.757 | 0.768 | 0.762 | 0.815 |
| Fold04.Rep1 | 0.854 | 0.820 | 0.850 | 0.777 | 0.798 | 0.783 | 0.854 |
| Fold05.Rep1 | 0.832 | 0.813 | 0.820 | 0.765 | 0.774 | 0.770 | 0.828 |
| Fold06.Rep1 | 0.823 | 0.795 | 0.823 | 0.755 | 0.766 | 0.753 | 0.817 |
| Fold07.Rep1 | 0.849 | 0.820 | 0.845 | 0.780 | 0.783 | 0.784 | 0.852 |
| Fold08.Rep1 | 0.813 | 0.788 | 0.818 | 0.746 | 0.756 | 0.750 | 0.816 |
| Fold09.Rep1 | 0.850 | 0.810 | 0.841 | 0.764 | 0.784 | 0.763 | 0.849 |
| Fold10.Rep1 | 0.840 | 0.825 | 0.817 | 0.786 | 0.791 | 0.790 | 0.842 |
| Fold01.Rep2 | 0.834 | 0.818 | 0.833 | 0.770 | 0.796 | 0.772 | 0.837 |
| Fold02.Rep2 | 0.838 | 0.814 | 0.823 | 0.781 | 0.786 | 0.783 | 0.839 |
| Fold03.Rep2 | 0.832 | 0.815 | 0.833 | 0.759 | 0.771 | 0.761 | 0.837 |
| Fold04.Rep2 | 0.841 | 0.809 | 0.844 | 0.753 | 0.758 | 0.750 | 0.837 |
| Fold05.Rep2 | 0.833 | 0.820 | 0.832 | 0.760 | 0.792 | 0.769 | 0.835 |
| Fold06.Rep2 | 0.840 | 0.798 | 0.838 | 0.753 | 0.765 | 0.760 | 0.838 |
| Fold07.Rep2 | 0.838 | 0.801 | 0.827 | 0.750 | 0.766 | 0.751 | 0.827 |
| Fold08.Rep2 | 0.819 | 0.791 | 0.808 | 0.763 | 0.785 | 0.762 | 0.827 |
| Fold09.Rep2 | 0.808 | 0.780 | 0.805 | 0.757 | 0.749 | 0.759 | 0.802 |
| Fold10.Rep2 | 0.847 | 0.817 | 0.827 | 0.759 | 0.791 | 0.761 | 0.834 |
| Fold01.Rep3 | 0.824 | 0.796 | 0.818 | 0.757 | 0.775 | 0.765 | 0.824 |
| Fold02.Rep3 | 0.836 | 0.808 | 0.825 | 0.757 | 0.778 | 0.757 | 0.833 |
| Fold03.Rep3 | 0.840 | 0.804 | 0.837 | 0.752 | 0.761 | 0.747 | 0.830 |
| Fold04.Rep3 | 0.821 | 0.806 | 0.829 | 0.764 | 0.767 | 0.766 | 0.828 |
| Fold05.Rep3 | 0.838 | 0.797 | 0.832 | 0.742 | 0.781 | 0.751 | 0.829 |
| Fold06.Rep3 | 0.823 | 0.788 | 0.814 | 0.756 | 0.776 | 0.757 | 0.820 |
| Fold07.Rep3 | 0.844 | 0.812 | 0.845 | 0.766 | 0.788 | 0.770 | 0.849 |
| Fold08.Rep3 | 0.860 | 0.828 | 0.856 | 0.799 | 0.813 | 0.801 | 0.860 |
| Fold09.Rep3 | 0.842 | 0.822 | 0.848 | 0.750 | 0.780 | 0.749 | 0.839 |
| Fold10.Rep3 | 0.835 | 0.817 | 0.825 | 0.771 | 0.792 | 0.771 | 0.838 |

**Supplementary Table 6.2 | Internal validation of the virtual biopsy system during cross-validation process (ah score)**

| Resample | Random Forest | Gradient Boosting Machine | Extreme Gradient Boosting Tree | Linear Discriminant Analysis | Model Averaged Neural Network | Multinomial logistic regression | Ensemble model |
|---|---|---|---|---|---|---|---|
| Fold01.Rep1 | 0.811 | 0.778 | 0.794 | 0.713 | 0.760 | 0.714 | 0.804 |
| Fold02.Rep1 | 0.785 | 0.769 | 0.755 | 0.736 | 0.713 | 0.733 | 0.800 |
| Fold03.Rep1 | 0.767 | 0.755 | 0.770 | 0.704 | 0.718 | 0.703 | 0.769 |
| Fold04.Rep1 | 0.769 | 0.753 | 0.769 | 0.703 | 0.719 | 0.706 | 0.773 |
| Fold05.Rep1 | 0.747 | 0.722 | 0.744 | 0.684 | 0.702 | 0.690 | 0.745 |
| Fold06.Rep1 | 0.780 | 0.754 | 0.757 | 0.701 | 0.753 | 0.710 | 0.778 |
| Fold07.Rep1 | 0.797 | 0.756 | 0.772 | 0.688 | 0.701 | 0.690 | 0.777 |
| Fold08.Rep1 | 0.743 | 0.720 | 0.747 | 0.686 | 0.700 | 0.689 | 0.745 |
| Fold09.Rep1 | 0.737 | 0.732 | 0.740 | 0.722 | 0.704 | 0.723 | 0.759 |
| Fold10.Rep1 | 0.754 | 0.737 | 0.751 | 0.698 | 0.717 | 0.708 | 0.762 |
| Fold01.Rep2 | 0.810 | 0.797 | 0.812 | 0.734 | 0.738 | 0.733 | 0.813 |
| Fold02.Rep2 | 0.770 | 0.746 | 0.771 | 0.693 | 0.690 | 0.692 | 0.763 |
| Fold03.Rep2 | 0.738 | 0.690 | 0.741 | 0.681 | 0.694 | 0.686 | 0.733 |
| Fold04.Rep2 | 0.755 | 0.710 | 0.725 | 0.707 | 0.736 | 0.714 | 0.756 |
| Fold05.Rep2 | 0.794 | 0.773 | 0.797 | 0.680 | 0.713 | 0.682 | 0.776 |
| Fold06.Rep2 | 0.752 | 0.741 | 0.763 | 0.701 | 0.724 | 0.708 | 0.760 |
| Fold07.Rep2 | 0.760 | 0.733 | 0.755 | 0.697 | 0.719 | 0.702 | 0.769 |
| Fold08.Rep2 | 0.781 | 0.752 | 0.782 | 0.707 | 0.722 | 0.706 | 0.780 |
| Fold09.Rep2 | 0.819 | 0.792 | 0.807 | 0.728 | 0.747 | 0.729 | 0.812 |
| Fold10.Rep2 | 0.784 | 0.776 | 0.777 | 0.703 | 0.734 | 0.707 | 0.784 |
| Fold01.Rep3 | 0.751 | 0.741 | 0.762 | 0.713 | 0.736 | 0.712 | 0.768 |
| Fold02.Rep3 | 0.786 | 0.759 | 0.784 | 0.695 | 0.712 | 0.700 | 0.779 |
| Fold03.Rep3 | 0.779 | 0.745 | 0.773 | 0.701 | 0.735 | 0.703 | 0.772 |
| Fold04.Rep3 | 0.752 | 0.732 | 0.748 | 0.693 | 0.699 | 0.693 | 0.758 |
| Fold05.Rep3 | 0.779 | 0.760 | 0.770 | 0.737 | 0.729 | 0.739 | 0.786 |
| Fold06.Rep3 | 0.780 | 0.737 | 0.764 | 0.672 | 0.684 | 0.682 | 0.755 |
| Fold07.Rep3 | 0.765 | 0.729 | 0.763 | 0.713 | 0.737 | 0.717 | 0.766 |
| Fold08.Rep3 | 0.799 | 0.785 | 0.788 | 0.698 | 0.732 | 0.692 | 0.795 |
| Fold09.Rep3 | 0.797 | 0.783 | 0.786 | 0.718 | 0.740 | 0.721 | 0.799 |
| Fold10.Rep3 | 0.773 | 0.737 | 0.757 | 0.685 | 0.706 | 0.684 | 0.764 |

**Supplementary Table 6.3 | Internal validation of the virtual biopsy system during cross-validation process (IFTA score)**

| Resample | Random Forest | Gradient Boosting Machine | Extreme Gradient Boosting Tree | Linear Discriminant Analysis | Model Averaged Neural Network | Multinomial logistic regression | Ensemble model |
|---|---|---|---|---|---|---|---|
| Fold01.Rep1 | 0.801 | 0.749 | 0.781 | 0.734 | 0.717 | 0.739 | 0.803 |
| Fold02.Rep1 | 0.869 | 0.832 | 0.855 | 0.793 | 0.763 | 0.790 | 0.855 |
| Fold03.Rep1 | 0.843 | 0.827 | 0.838 | 0.779 | 0.723 | 0.777 | 0.840 |
| Fold04.Rep1 | 0.790 | 0.764 | 0.768 | 0.728 | 0.748 | 0.735 | 0.796 |
| Fold05.Rep1 | 0.826 | 0.782 | 0.832 | 0.710 | 0.687 | 0.708 | 0.799 |
| Fold06.Rep1 | 0.862 | 0.832 | 0.857 | 0.763 | 0.734 | 0.766 | 0.851 |
| Fold07.Rep1 | 0.836 | 0.815 | 0.836 | 0.781 | 0.782 | 0.783 | 0.834 |
| Fold08.Rep1 | 0.848 | 0.839 | 0.856 | 0.761 | 0.768 | 0.768 | 0.847 |
| Fold09.Rep1 | 0.788 | 0.765 | 0.780 | 0.715 | 0.736 | 0.715 | 0.805 |
| Fold10.Rep1 | 0.852 | 0.851 | 0.862 | 0.741 | 0.808 | 0.749 | 0.853 |
| Fold01.Rep2 | 0.856 | 0.821 | 0.850 | 0.776 | 0.796 | 0.775 | 0.864 |
| Fold02.Rep2 | 0.836 | 0.819 | 0.841 | 0.767 | 0.729 | 0.763 | 0.837 |
| Fold03.Rep2 | 0.834 | 0.811 | 0.813 | 0.752 | 0.772 | 0.759 | 0.835 |
| Fold04.Rep2 | 0.769 | 0.731 | 0.777 | 0.690 | 0.709 | 0.695 | 0.764 |
| Fold05.Rep2 | 0.813 | 0.797 | 0.798 | 0.763 | 0.742 | 0.759 | 0.817 |
| Fold06.Rep2 | 0.786 | 0.762 | 0.789 | 0.732 | 0.732 | 0.733 | 0.801 |
| Fold07.Rep2 | 0.865 | 0.848 | 0.865 | 0.757 | 0.808 | 0.756 | 0.856 |
| Fold08.Rep2 | 0.826 | 0.817 | 0.835 | 0.753 | 0.803 | 0.761 | 0.842 |
| Fold09.Rep2 | 0.827 | 0.790 | 0.835 | 0.713 | 0.742 | 0.723 | 0.799 |
| Fold10.Rep2 | 0.875 | 0.853 | 0.856 | 0.810 | 0.831 | 0.807 | 0.876 |
| Fold01.Rep3 | 0.814 | 0.784 | 0.819 | 0.741 | 0.761 | 0.738 | 0.811 |
| Fold02.Rep3 | 0.834 | 0.813 | 0.828 | 0.750 | 0.738 | 0.752 | 0.835 |
| Fold03.Rep3 | 0.850 | 0.804 | 0.851 | 0.785 | 0.775 | 0.782 | 0.849 |
| Fold04.Rep3 | 0.859 | 0.823 | 0.856 | 0.783 | 0.788 | 0.781 | 0.858 |
| Fold05.Rep3 | 0.815 | 0.806 | 0.825 | 0.733 | 0.770 | 0.747 | 0.821 |
| Fold06.Rep3 | 0.837 | 0.810 | 0.837 | 0.740 | 0.749 | 0.750 | 0.828 |
| Fold07.Rep3 | 0.822 | 0.803 | 0.800 | 0.717 | 0.736 | 0.715 | 0.816 |
| Fold08.Rep3 | 0.774 | 0.753 | 0.785 | 0.741 | 0.736 | 0.740 | 0.791 |
| Fold09.Rep3 | 0.862 | 0.847 | 0.859 | 0.779 | 0.806 | 0.788 | 0.868 |
| Fold10.Rep3 | 0.839 | 0.811 | 0.838 | 0.725 | 0.732 | 0.725 | 0.833 |

**Supplementary Table 6.4 | Internal validation of the virtual biopsy system during cross-validation process (percentage of sclerotic glomeruli)**

| Resample | Random Forest | Gradient Boosting Machine | Extreme Gradient Boosting Tree | Model Averaged Neural Network | Ensemble model |
|---|---|---|---|---|---|
| Fold01.Rep1 | 6.050 | 6.573 | 6.043 | 6.714 | 6.044 |
| Fold02.Rep1 | 5.734 | 6.257 | 5.662 | 6.479 | 5.969 |
| Fold03.Rep1 | 5.606 | 6.288 | 5.503 | 6.313 | 5.974 |
| Fold04.Rep1 | 5.624 | 6.447 | 5.762 | 6.548 | 6.014 |
| Fold05.Rep1 | 5.797 | 6.559 | 5.806 | 6.760 | 5.997 |
| Fold06.Rep1 | 5.889 | 6.529 | 5.649 | 6.483 | 5.998 |
| Fold07.Rep1 | 5.591 | 6.344 | 5.667 | 6.535 | 6.029 |
| Fold08.Rep1 | 5.905 | 6.524 | 5.945 | 6.492 | 5.981 |
| Fold09.Rep1 | 5.995 | 6.733 | 5.991 | 6.670 | 5.981 |
| Fold10.Rep1 | 6.084 | 6.636 | 5.896 | 6.680 | 6.002 |
| Fold01.Rep2 | 6.109 | 6.609 | 5.978 | 6.699 | 5.935 |
| Fold02.Rep2 | 5.592 | 6.237 | 5.450 | 6.281 | 6.048 |
| Fold03.Rep2 | 5.551 | 6.312 | 5.475 | 6.465 | 5.992 |
| Fold04.Rep2 | 5.813 | 6.389 | 5.856 | 6.477 | 6.005 |
| Fold05.Rep2 | 5.644 | 6.399 | 5.719 | 6.599 | 6.035 |
| Fold06.Rep2 | 5.922 | 6.780 | 5.821 | 6.865 | 5.984 |
| Fold07.Rep2 | 5.918 | 6.629 | 5.928 | 6.793 | 5.987 |
| Fold08.Rep2 | 5.907 | 6.533 | 5.702 | 6.549 | 6.030 |
| Fold09.Rep2 | 5.730 | 6.476 | 5.686 | 6.582 | 6.004 |
| Fold10.Rep2 | 5.670 | 6.486 | 5.699 | 6.451 | 5.970 |
| Fold01.Rep3 | 6.143 | 6.830 | 6.097 | 6.873 | 5.973 |
| Fold02.Rep3 | 5.938 | 6.678 | 5.835 | 6.751 | 5.996 |
| Fold03.Rep3 | 5.641 | 6.336 | 5.594 | 6.394 | 5.970 |
| Fold04.Rep3 | 5.792 | 6.418 | 5.718 | 6.508 | 6.033 |
| Fold05.Rep3 | 5.768 | 6.391 | 5.800 | 6.493 | 6.058 |
| Fold06.Rep3 | 5.909 | 6.636 | 5.891 | 6.711 | 6.040 |
| Fold07.Rep3 | 5.877 | 6.508 | 5.845 | 6.627 | 5.959 |
| Fold08.Rep3 | 5.494 | 6.248 | 5.494 | 6.269 | 6.016 |
| Fold09.Rep3 | 6.031 | 6.617 | 6.040 | 6.825 | 6.009 |
| Fold10.Rep3 | 5.473 | 6.174 | 5.494 | 6.306 | 5.937 |

**Supplementary Table 7 | Calibration confusion matrices of the virtual biopsy system (internal validation)**

**Supplementary Table 7.1 | Confusion matrix on the arteriosclerosis (cv biopsy lesion score)**

| Reference | Virtual Biopsy Prediction | | | |
|---|---|---|---|---|
| | 0 | 1 | 2 | 3 |
| 0 | 5382 | 1255 | 327 | 109 |
| 1 | 945 | 1481 | 568 | 111 |
| 2 | 147 | 340 | 741 | 97 |
| 3 | 4 | 20 | 94 | 134 |

**Supplementary Table 7.2 | Confusion matrix on the arteriolar hyalinosis (ah biopsy lesion score)**

| Reference | Virtual Biopsy Prediction | | | |
|---|---|---|---|---|
| | 0 | 1 | 2 | 3 |
| 0 | 6286 | 1292 | 361 | 303 |
| 1 | 875 | 1118 | 395 | 158 |
| 2 | 182 | 292 | 381 | 113 |
| 3 | 21 | 39 | 58 | 99 |

**Supplementary Table 7.3 | Confusion matrix on the interstitial fibrosis and tubular atrophy (IFTA biopsy lesion score)**

| Reference | Virtual Biopsy Prediction | | | |
|---|---|---|---|---|
| | 0 | 1 | 2 | 3 |
| 0 | 6071 | 1475 | 230 | 46 |
| 1 | 1086 | 2175 | 319 | 67 |
| 2 | 41 | 198 | 286 | 37 |
| 3 | 8 | 21 | 27 | 61 |

**Supplementary Table 8 | Comparison of donor baseline characteristics between the derivation cohort and the external validation cohort**

| | N | Overall (n=14,032) | N | Derivation (n=12,402) | N | External (n=1,630) | p-value |
|---|---|---|---|---|---|---|---|
| Age (years), mean (SD) | 14032 | 46.8 (14.7) | 12402 | 46.7 (14.9) | 1630 | 48.0 (13.2) | <0.001 |
| Sex female, No. (%) | 14021 | 6173 (44.0%) | 12391 | 5450 (44.0%) | 1630 | 723 (44.4%) | 0.796 |
| Donor type | | | | | | | |
| Deceased donor, No. (%) | 14032 | 10519 (75.0%) | 12402 | 9395 (75.8%) | 1630 | 1124 (69.0%) | <0.001 |
| Death from circulatory disease, No. (%)* | 10484 | 1602 (15.3%) | 9360 | 1471 (15.7%) | 1124 | 131 (11.7%) | <0.001 |
| Death from cerebrovascular disease, No. (%)* | 10478 | 4526 (43.2%) | 9354 | 4001 (42.8%) | 1124 | 525 (46.7%) | 0.0130 |
| Diabetes mellitus, No. (%) | 12200 | 997 (8.2%) | 10585 | 782 (7.4%) | 1615 | 215 (13.3%) | <0.001 |
| Hypertension, No. (%) | 12892 | 2969 (23.0%) | 11274 | 2375 (21.1%) | 1618 | 594 (36.7%) | <0.001 |
| BMI (kg/$m^2$), mean (SD) | 13080 | 27.0 (5.6) | 11456 | 26.9 (5.5) | 1624 | 27.6 (6.3) | <0.001 |
| HCV status, No. (%) | 13634 | 282 (2.1%) | 12004 | 233 (1.9%) | 1630 | 49 (3.0%) | 0.00610 |
| Creatinine (mg/dL), mean (SD) | 12141 | 1.2 (1.0) | 10924 | 1.2 (1.0) | 1217 | 1.6 (1.1) | <0.001 |
| Proteinuria, No. (%) | 10841 | 2491 (23.0%) | 9218 | 1904 (20.7%) | 1623 | 587 (36.2%) | <0.001 |
| Number of Glomeruli, mean (SD) | 7964 | 41.2 (35.5) | 6993 | 39.3 (33.5) | 971 | 54.7 (45.0) | <0.001 |
| Arteriosclerosis (cv) Banff score, No. (%) | 13380 | | 11755 | | 1625 | | <0.001 |
| 0 | | 7526 (56.2%) | | 7073 (60.2%) | | 453 (27.9%) | |
| 1 | | 3656 (27.3%) | | 3105 (26.4%) | | 551 (33.9%) | |
| 2 | | 1915 (14.3%) | | 1325 (11.3%) | | 590 (36.3%) | |
| 3 | | 283 (2.1%) | | 252 (2.1%) | | 31 (1.9%) | |
| Arteriolar hyalinosis (ah) Banff score, No. (%) | 12926 | | 11973 | | 953 | | <0.001 |
| 0 | | 8755 (67.7%) | | 8242 (68.8%) | | 513 (53.8%) | |
| 1 | | 2912 (22.5%) | | 2546 (21.3%) | | 366 (38.4%) | |
| 2 | | 1029 (8.0%) | | 968 (8.1%) | | 61 (6.4%) | |
| 3 | | 230 (1.8%) | | 217 (1.8%) | | 13 (1.4%) | |
| Interstitial fibrosis and tubular atrophy (IFTA) Banff score, No. (%) | 13778 | | 12148 | | 1630 | | <0.001 |
| 0 | | 8480 (61.5%) | | 7822 (64.4%) | | 658 (40.4%) | |
| 1 | | 4148 (30.1%) | | 3647 (30.0%) | | 501 (30.7%) | |
| 2 | | 1029 (7.5%) | | 562 (4.6%) | | 467 (28.7%) | |
| 3 | | 121 (0.9%) | | 117 (1.0%) | | 4 (0.2%) | |
| Glomerulosclerosis, median (interquartile range) | 10455 | 3.0 (0.0-11.0) | 8826 | 3.0 (0.0-10.0) | 1629 | 2.1 (0.0-12.5) | <0.001 |

**Supplementary Table 9 | Baseline donor characteristics of the external validation cohort by center**

| | Overall (n=1,630) | Columbia University Medical Center (n=1,332) | Sun-Yat sen university (n=298) |
|---|---|---|---|
| Age (years), mean (SD) | 48.0 (13.2) | 48.9 (13.3) | 44.1 (11.5) |
| Sex female, No. (%) | 723 (44.4%) | 628 (47.1%) | 95 (31.9%) |
| Donor type | | | |
| Deceased donor, No. (%) | 1124 (69.0%) | 920 (69.1%) | 204 (68.5%) |
| Death from circulatory disease, No. (%)* | 131 (11.7%) | 94 (10.2%) | 37 (18.1%) |
| Death from cerebrovascular disease, No. (%)* | 525 (46.7%) | 472 (51.3%) | 53 (26.0%) |
| Diabetes mellitus, No. (%) | 215 (13.3%) | 208 (15.8%) | 7 (2.3%) |
| Hypertension, No. (%) | 594 (36.7%) | 558 (42.3%) | 36 (12.1%) |
| BMI (kg/$m^2$), mean (SD) | 27.6 (6.3) | 28.6 (6.4) | 23.1 (2.8) |
| HCV status, No. (%) | 49 (3.0%) | 44 (3.3%) | 5 (1.7%) |
| Creatinine (mg/dL), mean (SD) | 1.6 (1.1) | 1.7 (1.1) | 1.3 (0.9) |
| Proteinuria, No. (%) | 587 (36.2%) | 480 (36.2%) | 107 (35.9%) |
| Number of Glomeruli, mean (SD) | 54.7 (45.0) | 71.1 (44.9) | 17.7 (7.9) |
| Arteriosclerosis (cv) Banff score, No. (%) | | | |
| 0 | 453 (27.9%) | 304 (22.9%) | 149 (50.3%) |
| 1 | 551 (33.9%) | 412 (31.0%) | 139 (47.0%) |
| 2 | 590 (36.3%) | 582 (43.8%) | 8 (2.7%) |
| 3 | 31 (1.9%) | 31 (2.3%) | 0 (0.0%) |
| Arteriolar hyalinosis (ah) Banff score, No. (%) | | | |
| 0 | 513 (53.8%) | 328 (50.1%) | 185 (62.1%) |
| 1 | 366 (38.4%) | 274 (41.8%) | 92 (30.9%) |
| 2 | 61 (6.4%) | 44 (6.7%) | 17 (5.7%) |
| 3 | 13 (1.4%) | 9 (1.4%) | 4 (1.3%) |
| Interstitial fibrosis and tubular atrophy (IFTA) Banff score, No. (%) | | | |
| 0 | 658 (40.4%) | 532 (39.9%) | 126 (42.3%) |
| 1 | 501 (30.7%) | 338 (25.4%) | 163 (54.7%) |
| 2 | 467 (28.7%) | 459 (34.5%) | 8 (2.7%) |
| 3 | 4 (0.2%) | 3 (0.2%) | 1 (0.3%) |
| Glomerulosclerosis, median (interquartile range) | 2.1 (0.0-12.5) | 2.0 (0.0-13.8) | 2.9 (0.0-9.1) |

## Supplementary Table 10 | Baseline donor characteristics of the external validation cohort by donor type

| | N | Overall (n=1,630) | N | Deceased Donor (n=1,124) | N | Living Donor (n=506) | p-value |
|---|---|---|---|---|---|---|---|
| Age (years), mean (SD) | 1630 | 48.0 (13.2) | 1124 | 49.9 (13.5) | 506 | 43.7 (11.3) | <0.001 |
| Sex female, No. (%) | 1630 | 723 (44.4%) | 1124 | 428 (38.1%) | 506 | 295 (58.3%) | <0.001 |
| Donor type | | | | | | | |
| Deceased donor, No. (%) | 1630 | 1124 (69.0%) | 1124 | 1124 (100.0%) | 506 | 0 (0.0%) | <0.001 |
| Death from circulatory disease, No. (%)* | 1124 | 131 (11.7%) | 1124 | 131 (11.7%) | 0 | 0 (-) | <0.001 |
| Death from cerebrovascular disease, No. (%)* | 1124 | 525 (46.7%) | 1124 | 525 (46.7%) | 0 | 0 (-) | <0.001 |
| Diabetes mellitus, No. (%) | 1615 | 215 (13.3%) | 1109 | 215 (19.4%) | 506 | 0 (0.0%) | <0.001 |
| Hypertension, No. (%) | 1618 | 594 (36.7%) | 1112 | 594 (53.4%) | 506 | 0 (0.0%) | <0.001 |
| BMI (kg/$m^2$), mean (SD) | 1624 | 27.6 (6.3) | 1123 | 28.4 (6.9) | 501 | 25.8 (4.3) | <0.001 |
| HCV status, No. (%) | 1630 | 49 (3.0%) | 1124 | 48 (4.3%) | 506 | 1 (0.2%) | <0.001 |
| Creatinine (mg/dL), mean (SD) | 1217 | 1.6 (1.1) | 1123 | 1.7 (1.1) | 94 | 0.8 (0.2) | <0.001 |
| Proteinuria, No. (%) | 1623 | 587 (36.2%) | 1117 | 587 (52.6%) | 506 | 0 (0.0%) | <0.001 |
| Number of Glomeruli, mean (SD) | 971 | 54.7 (45.0) | 877 | 59.1 (45.2) | 94 | 14.1 (4.3) | <0.001 |
| Arteriosclerosis (cv) Banff score, No. (%) | 1625 | | 1123 | | 502 | | <0.001 |
| 0 | | 453 (27.9%) | | 249 (22.2%) | | 204 (40.6%) | |
| 1 | | 551 (33.9%) | | 306 (27.2%) | | 245 (48.8%) | |
| 2 | | 590 (36.3%) | | 537 (47.8%) | | 53 (10.6%) | |
| 3 | | 31 (1.9%) | | 31 (2.8%) | | 0 (0.0%) | |
| Arteriolar hyalinosis (ah) Banff score, No. (%) | 953 | | 447 | | 506 | | <0.001 |
| 0 | | 513 (53.8%) | | 198 (44.3%) | | 315 (62.3%) | |
| 1 | | 366 (38.4%) | | 176 (39.4%) | | 190 (37.5%) | |
| 2 | | 61 (6.4%) | | 60 (13.4%) | | 1 (0.2%) | |
| 3 | | 13 (1.4%) | | 13 (2.9%) | | 0 (0.0%) | |
| Interstitial fibrosis and tubular atrophy (IFTA) Banff score, No. (%) | 1630 | | 1124 | | 506 | | <0.001 |
| 0 | | 658 (40.4%) | | 287 (25.5%) | | 371 (73.3%) | |
| 1 | | 501 (30.7%) | | 366 (32.6%) | | 135 (26.7%) | |
| 2 | | 467 (28.7%) | | 467 (41.5%) | | 0 (0.0%) | |
| 3 | | 4 (0.2%) | | 4 (0.4%) | | 0 (0.0%) | |
| Glomerulosclerosis, median (interquartile range) | 1629 | 2.1 (0.0-12.5) | 1123 | 9.7 (<0.1-14.4) | 506 | 0.0 (0.0-0.1) | <0.001 |

**Supplementary Table 11 | Calibration confusion matrices of the virtual biopsy system (external validation)**

**Supplementary Table 11.1 | Confusion matrix on the arteriosclerosis (cv biopsy lesion score)**

| Reference (external cohort) | Virtual Biopsy Prediction | | | |
|---|---|---|---|---|
| | **0** | **1** | **2** | **3** |
| **0** | 301 | 120 | 20 | 12 |
| **1** | 256 | 202 | 71 | 22 |
| **2** | 56 | 132 | 217 | 185 |
| **3** | 3 | 6 | 10 | 12 |

**Supplementary Table 11.2 | Confusion matrix on the arteriolar hyalinosis (ah biopsy lesion score)**

| Reference (external cohort) | Virtual Biopsy Prediction | | | |
|---|---|---|---|---|
| | **0** | **1** | **2** | **3** |
| **0** | 448 | 43 | 20 | 2 |
| **1** | 266 | 54 | 30 | 16 |
| **2** | 13 | 13 | 21 | 14 |
| **3** | 4 | 2 | 3 | 4 |

**Supplementary Table 11.3 | Confusion matrix on the interstitial fibrosis and tubular atrophy (IFTA biopsy lesion score)**

| Reference (external cohort) | Virtual Biopsy Prediction | | | |
|---|---|---|---|---|
| | **0** | **1** | **2** | **3** |
| **0** | 546 | 102 | 9 | 1 |
| **1** | 225 | 200 | 64 | 12 |
| **2** | 6 | 236 | 191 | 34 |
| **3** | 0 | 3 | 1 | 0 |

**Supplementary Table 12 | Internal validation of the virtual biopsy system in various subpopulations and clinical scenarios**

| Subpopulations / Clinical Scenario | | Discrimination | | | |
|---|---|---|---|---|---|
| | | Arteriosclerosis (cv)* | Arteriolar hyalinosis (ah)* | Interstitial fibrosis and tubular atrophy (IFTA)* | Glomerulosclerosis† |
| **Region** | Europe | 0.767 | 0.711 | 0.742 | 7.393 |
| | North America | 0.883 | 0.820 | 0.884 | 4.915 |
| | Australia | 0.760 | 0.693 | 0.837 | 5.522 |
| **Ethnicity‡** | African American | 0.949 | 0.861 | 0.912 | 4.421 |
| | Caucasian | 0.837 | 0.782 | 0.875 | 6.454 |
| | Others§ | 0.903 | 0.882 | 0.909 | 4.711 |
| **Donor criteria** | Extended criteria donors (ECD) | 0.811 | 0.769 | 0.801 | 9.437 |
| | Standard criteria donors (SCD) OR living donors | 0.834 | 0.758 | 0.816 | 4.455 |
| **Biopsy type** | Preimplantation (before anastomosis) | 0.844 | 0.784 | 0.842 | 6.455 |
| | Postreperfusion (after anastomosis) | 0.785 | 0.758 | 0.679 | 5.225 |

**Supplementary Table 13 | KDPI as a parameter in predicting day-zero kidney biopsy results**

| Model | Cohort | Validation | Multi-AUC | | | Mean Absolute Error |
|---|---|---|---|---|---|---|
| | | | cv | ah | IFTA | Glomerulosclerosis |
| KDPI | Internal | Cross-validation | 0.688 | 0.644 | 0.716 | 6.647 |
| | External | Columbia University | 0.625 | 0.668 | 0.638 | 4.947 |
| | | Sun Yat-sen University | 0.659 | 0.552 | 0.710 | 4.193 |

**Supplementary Table 14 | Summary of participating centers' biopsy practices and procedures**

| Country | Center | Performed | Timing | Technique | Tissue processing | Tissue stain | Interpretation |
|---|---|---|---|---|---|---|---|
| France | PTG (Necker)(SLS)(Toulouse) | Surgeon | Preimplantation (before anastomosis) | Core needle | alcohol–formalin–acetic acid fixed (AFA) and paraffin-embedded (PE) | Periodic acid-Schiff (PAS), Masson's trichrome, Hematoxylin and eosin stain, Jones (methenamine silver) | Renal pathologist |
| France | Kremlin Bicêtre | Surgeon | Preimplantation (before anastomosis) | Wedge, Core needle | frozen, formalin-fixed paraffin-embedded (FFPE) | Periodic acid-Schiff (PAS), Masson's trichrome, Hematoxylin and eosin stain, Jones (methenamine silver) | General pathologist, Renal pathologist |
| Belgium | UZ Leuven | Surgeon | Preimplantation (before anastomosis) | Core needle | frozen, formalin-fixed paraffin-embedded (FFPE) | Periodic acid-Schiff (PAS), Masson's trichrome, Hematoxylin and eosin stain, Jones (methenamine silver) | Renal pathologist |
| Belgium | CHU Liège | Surgeon | Postreperfusion (after the anastomosis) | Wedge | formalin-fixed paraffin-embedded (FFPE) | Periodic acid-Schiff (PAS), Masson's trichrome, Hematoxylin and eosin stain | Renal pathologist |
| Croatia | University hospital centre Zagreb | Surgeon | Preimplantation (before anastomosis) | Wedge | formalin-fixed paraffin-embedded (FFPE) | Periodic acid-Schiff (PAS), Hematoxylin and eosin stain, Mallory-Weiss stain | Renal pathologist |
| Spain | Hospital Clinic i Provincial de Barcelona | Surgeon | Preimplantation (before anastomosis) | Wedge | frozen | Hematoxylin and eosin stain | General pathologist |
| Spain | Vall d'Hebron University Hospital | Surgeon | Preimplantation (before anastomosis) | Wedge | formalin-fixed paraffin-embedded (FFPE) | Hematoxylin and eosin stain | General pathologist, Renal pathologist |
| Spain | Hospital Universitari de Bellvitge | Surgeon | Preimplantation (before anastomosis) | Wedge, Core needle | frozen | Periodic acid-Schiff (PAS) | General pathologist |

**Supplementary Table 14 | Summary of participating centers' biopsy practices and procedures (continued)**

| Country | Center | Performed | Timing | Technique | Tissue processing | Tissue stain | Interpretation |
|---|---|---|---|---|---|---|---|
| Canada | University of Alberta | Surgeon | Postreperfusion (after the anastomosis) | Wedge, Core needle | frozen, formalin-fixed paraffin-embedded (FFPE) | Periodic acid-Schiff (PAS), Masson's trichrome, Hematoxylin and eosin stain | Renal pathologist |
| Canada | University of British Columbia | Surgeon | Postreperfusion (after the anastomosis) | Core needle | formalin-fixed paraffin-embedded (FFPE) | Periodic acid-Schiff (PAS), Masson's trichrome, Hematoxylin and eosin stain | Renal pathologist |
| United States | Columbia University Medical Center | Surgeon | Postreperfusion (after the anastomosis) | Core needle | formalin-fixed paraffin-embedded (FFPE) | Periodic acid-Schiff (PAS), Masson's trichrome, Hematoxylin and eosin stain, Jones (methenamine silver) | Renal pathologist |
| United States | Mayo Clinic | Surgeon | Postreperfusion (after the anastomosis) | Core needle | formalin-fixed paraffin-embedded (FFPE) | Periodic acid-Schiff (PAS), Hematoxylin and eosin stain | Renal pathologist |
| United States | OneLegacy | Surgeon | Preimplantation (before anastomosis) | Wedge | frozen, formalin-fixed paraffin-embedded (FFPE) | Periodic acid-Schiff (PAS), Masson's trichrome, Hematoxylin and eosin stain, Jones (methenamine silver) | Renal pathologist |
| Australia | Royal Adelaide Hospital | Surgeon | Postreperfusion (after the anastomosis) | Wedge | formalin-fixed paraffin-embedded (FFPE) | Periodic acid-Schiff (PAS), Hematoxylin and eosin stain | Renal pathologist |
| China | Sun Yat-sen University | Surgeon | Preimplantation (before anastomosis) | Wedge, Core needle | formalin-fixed paraffin-embedded (FFPE) | Periodic acid-Schiff (PAS), Masson's trichrome, Hematoxylin and eosin stain, Jones (methenamine silver) | Renal pathologist |

**Supplementary Table 15 | Summary of kidney day-zero histological lesion scores (international Banff classification grading scheme)**

| Banff lesion score | Abbreviation | Grading 0 | Grading 1 | Grading 2 | Grading 3 |
|---|---|---|---|---|---|
| Vascular fibrous intimal thickening | cv | None | <=25% | 26-50% | >50% |
| Arteriolar hyalinosis | ah | None | Mild to moderate in >=1 | Moderate to severe in >1 | Severe in many |
| Interstitial fibrosis | ci | <=5% | 6-25% | 26-50% | >50% |
| Tubular atrophy | ct | None | <=25% | 26-50% | >50% |

# 3. SUPPLEMENTARY FIGURES

**Supplementary Figure 1 | Virtual biopsy system usage in real-life situation and organ allocation**

**Real clinical case A:**

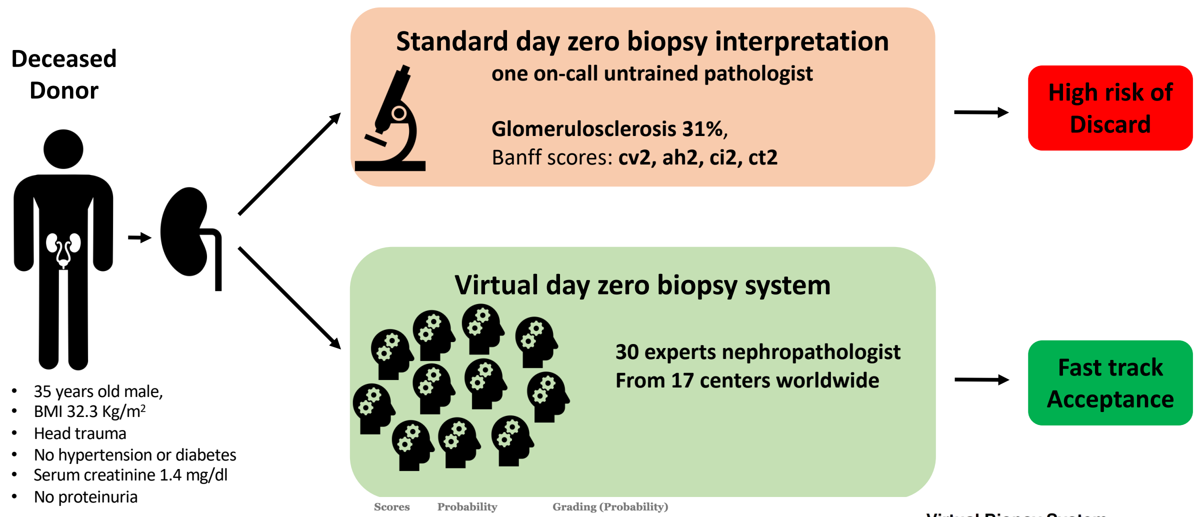


| Scores | Probability | Grading (Probability) |
|---|---|---|
| *Arteriosclerosis (cv Banff score)* | | |
| 0 | 0.776 | None / Mild (0.776+0.137=0.913) |
| 1 | 0.137 | |
| 2 | 0.047 | Moderate / Severe (0.047+0.04=0.087) |
| 3 | 0.04 | |
| *Arteriolar hyalinosis (ah Banff score)* | | |
| 0 | 0.67 | None / Mild (0.67+0.167=0.837) |
| 1 | 0.167 | |
| 2 | 0.125 | Moderate / Severe (0.125+0.038=0.163) |
| 3 | 0.038 | |
| *Interstitial fibrosis and tubular atrophy (IFTA Banff score)* | | |
| 0 | 0.746 | None / Mild (0.746+0.171=0.917) |
| 1 | 0.171 | |
| 2 | 0.061 | Moderate / Severe (0.061+0.022=0.083) |
| 3 | 0.022 | |
| *Glomerulosclerosis (% of sclerotic glomeruli)* | | |
| 6.646% | | |

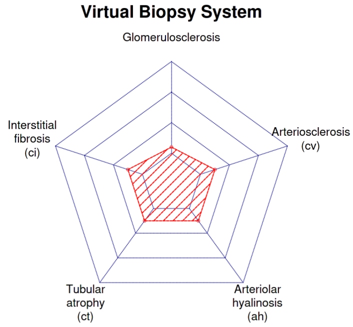


**Real clinical case A:** in this case based on real life data of a 35-year-old male donor with obesity who died from a head trauma, the day-zero biopsy was performed and interpreted in real life setting by non-nephropathologist showed 31% of glomerulosclerosis, moderate arteriosclerosis (cv2 Banff score), moderate arteriolar hyalinosis (ah2 Banff score) and moderate interstitial fibrosis and tubular atrophy (ci2 and ct2 Banff scores). These results lead to the decision to discard the kidney. The virtual biopsy system based on donor characteristics showed a high probability of None/mild arteriosclerosis (cv0/1 Banff score), None/mild arteriolar hyalinosis (ah0/1 Banff score), None/mild interstitial fibrosis and tubular atrophy (ci0/1 and ct0/1 Banff scores) and a percentage of glomerulosclerosis of 6.646%. In this case, the virtual biopsy might help to not discard the kidney for histological reason.

**Real clinical case B:**

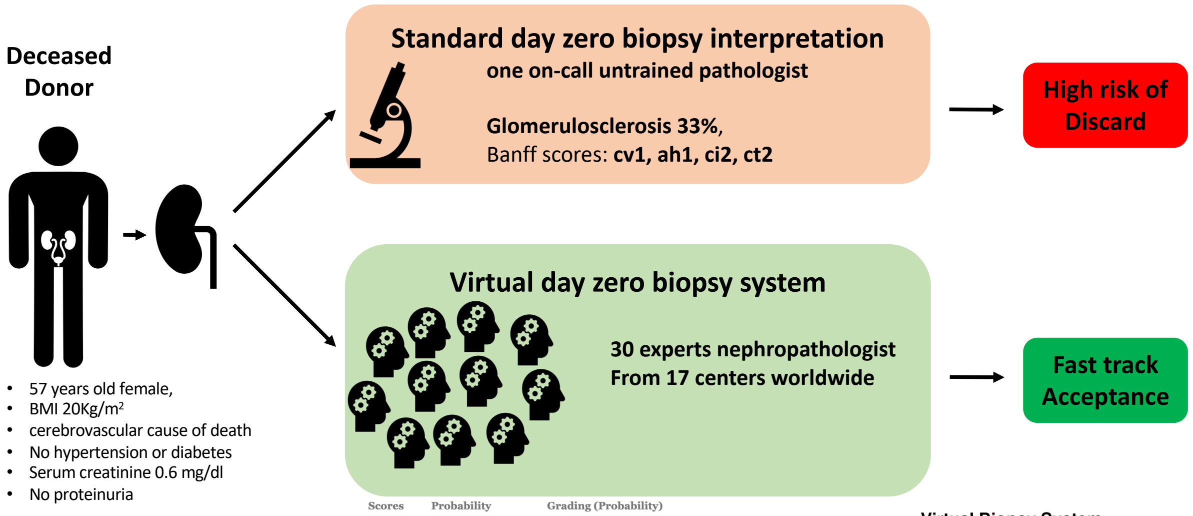


| Scores | Probability | Grading (Probability) |
|---|---|---|
| *Arteriosclerosis (cv Banff score)* | | |
| 0 | 0.433 | None / Mild (0.433+0.251=0.684) |
| 1 | 0.251 | |
| 2 | 0.242 | Moderate / Severe (0.242+0.074=0.316) |
| 3 | 0.074 | |
| *Arteriolar hyalinosis (ah Banff score)* | | |
| 0 | 0.226 | None / Mild (0.226+0.462=0.688) |
| 1 | 0.462 | |
| 2 | 0.179 | Moderate / Severe (0.179+0.133=0.312) |
| 3 | 0.133 | |
| *Interstitial fibrosis and tubular atrophy (IFTA Banff score)* | | |
| 0 | 0.298 | None / Mild (0.298+0.431=0.729) |
| 1 | 0.431 | |
| 2 | 0.215 | Moderate / Severe (0.215+0.056=0.271) |
| 3 | 0.056 | |
| *Glomerulosclerosis (% of sclerotic glomeruli)* | | |
| 7.558% | | |

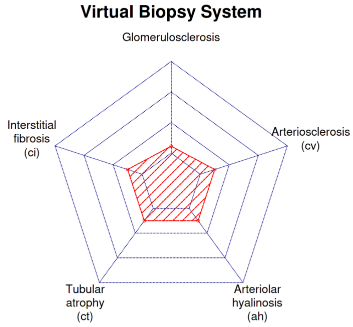


**Real clinical case B:** in this case based on real life data of a 57-year-old female donor who died from a stroke, the day zero biopsy performed and interpreted in the real life setting by non-nephropathologists showed 33 % of glomerulosclerosis, mild arteriosclerosis (cv1 Banff score), mild arteriolar hyalinosis (ah1 Banff score) and moderate to severe interstitial fibrosis and tubular atrophy (ci2 and ct2 Banff scores). These results lead to the decision to discard the kidney. The virtual biopsy system based on donor characteristics showed a high probability of None/mild arteriosclerosis (cv0/1 Banff score), None/mild arteriolar hyalinosis (ah0/1 Banff score), None/mild interstitial fibrosis and tubular atrophy (ci0/1 and ct0/1 Banff scores) and a percentage of glomerulosclerosis of 7.558%. In this case the virtual biopsy might help to not discard the kidney for histological reasons.

**Supplementary Figure 2 | The potential utility and impact of the virtual biopsy system for organ allocation**

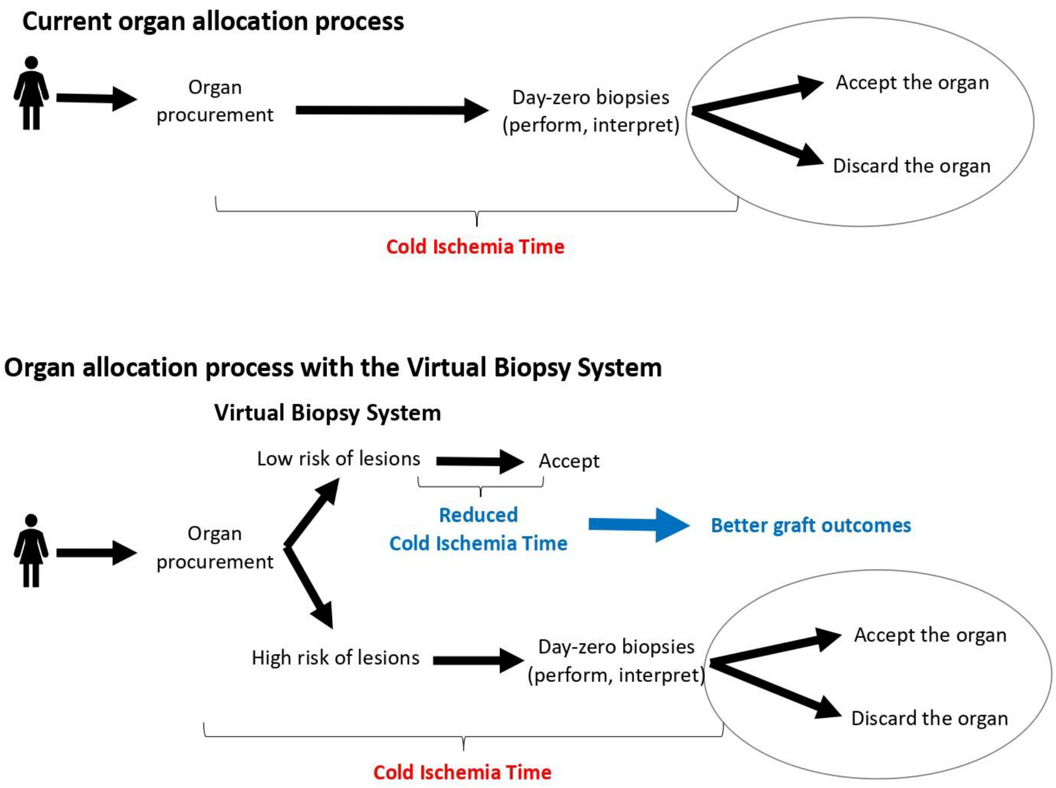

## 4. ADDITIONAL SUPPLEMENTARY MATERIALS

**Supplementary Movie 1 | Video of the online application**

## 5. SUPPLEMENTARY REFERENCES


1. Solez, K. *et al.* International standardization of criteria for the histologic diagnosis of renal allograft rejection: the Banff working classification of kidney transplant pathology. *Kidney Int* **44**, 411–422 (1993).
2. Solez, K. *et al.* Banff 07 classification of renal allograft pathology: updates and future directions. *Am J Transplant* **8**, 753–760 (2008).
3. Liapis, H. *et al.* Banff Histopathological Consensus Criteria for Preimplantation Kidney Biopsies. *Am J Transplant* **17**, 140–150 (2017).
4. Solez, K. *et al.* Banff '05 Meeting Report: differential diagnosis of chronic allograft injury and elimination of chronic allograft nephropathy ('CAN'). *Am J Transplant* **7**, 518–526 (2007).
5. Stekhoven, D. J. & Bühlmann, P. MissForest—non-parametric missing value imputation for mixed-type data. *Bioinformatics* **28**, 112–118 (2012).
6. Hand, D. J. & Till, R. J. A Simple Generalisation of the Area Under the ROC Curve for Multiple Class Classification Problems. *Machine Learning* **45**, 171–186 (2001).
