# Supplementary material for: A Machine Learning-Driven Virtual Biopsy System For Kidney Transplant Patients
Source: Nat Commun. 2024 Jan 16;15:554. doi: 10.1038/s41467-023-44595-z (PMC10791605; doi:10.1038/s41467-023-44595-z)
Supplement: Supplementary file 3 — Description of Additional Supplementary Files [file 41467_2023_44595_MOESM3_ESM.pdf]

File name: Supplementary Movie 1

Description: Virtual Biopsy System application. Available online: https://transplantpredictionsystem.shinyapps.io/Virtual_Biopsy_System
