# Supplementary material for: A Machine Learning-Driven Virtual Biopsy System For Kidney Transplant Patients
Source: Nat Commun. 2024 Jan 16;15:554. doi: 10.1038/s41467-023-44595-z (PMC10791605; doi:10.1038/s41467-023-44595-z)
Supplement: Supplementary file 5 — Reporting Summary [file 41467_2023_44595_MOESM5_ESM.pdf]

nature portfolio

Corresponding author(s): Alexandre Loupy (MD, PhD)

Last updated by author(s): Oct 23, 2023

Reporting Summary

Nature Portfolio wishes to improve the reproducibility of the work that we publish. This form provides structure for consistency and transparency in reporting. For further information on Nature Portfolio policies, see our Editorial Policies and the Editorial Policy Checklist.

Statistics

For all statistical analyses, confirm that the following items are present in the figure legend, table legend, main text, or Methods section.

n/a	Confirmed	
☐	☒	The exact sample size (*n*) for each experimental group/condition, given as a discrete number and unit of measurement
☐	☒	A statement on whether measurements were taken from distinct samples or whether the same sample was measured repeatedly
☐	☒	The statistical test(s) used AND whether they are one- or two-sided<br>*Only common tests should be described solely by name; describe more complex techniques in the Methods section.*
☐	☒	A description of all covariates tested
☐	☒	A description of any assumptions or corrections, such as tests of normality and adjustment for multiple comparisons
☐	☒	A full description of the statistical parameters including central tendency (e.g. means) or other basic estimates (e.g. regression coefficient) AND variation (e.g. standard deviation) or associated estimates of uncertainty (e.g. confidence intervals)
☐	☒	For null hypothesis testing, the test statistic (e.g. *F*, *t*, *r*) with confidence intervals, effect sizes, degrees of freedom and *P* value noted<br>*Give P values as exact values whenever suitable.*
☒	☐	For Bayesian analysis, information on the choice of priors and Markov chain Monte Carlo settings
☒	☐	For hierarchical and complex designs, identification of the appropriate level for tests and full reporting of outcomes
☒	☐	Estimates of effect sizes (e.g. Cohen's *d*, Pearson's *r*), indicating how they were calculated

Our web collection on statistics for biologists contains articles on many of the points above.

Software and code

Policy information about availability of computer code

Data collection	R (version 3.5.1)
Data analysis	Softwares: R software (version 3.5.1), RStudio software (version 2022.7.2.576). Packages: randomForest (version 4.6-14), gbm (version 2.1.5), xgboost (version 1.4.1.1), plyr (version 1.8.4), MASS (version 7.3-51.4), nnet (version 7.3-12), caret (version 6.0-84), caretEnsemble (version 2.0.1), tidyverse (version 1.3.0), ggsci (version 2.9), rsample (version 0.1.1), tidymodels (version 0.0.2), patchwork (version 1.0.0), dplyr (version 1.0.7), ggplot2 (version 3.3.1), yardstick (version 0.0.8), readr (version 1.3.1), cvms (version 1.3.3), pROC (version 1.18.0), rlist (version 0.4.6.2), autoxgboost (version 0.0.0.9000), shiny (version 1.6.0), shinythemes (version 1.1.2), kableExtra (version 1.3.4), and compareGroups (version 4.0.0). Complete code to reproduce the figures is available in the synapse public repository (https://www.synapse.org/VirtualBiopsySystem). A sign-in process is required to access the code.

For manuscripts utilizing custom algorithms or software that are central to the research but not yet described in published literature, software must be made available to editors and reviewers. We strongly encourage code deposition in a community repository (e.g. GitHub). See the Nature Portfolio guidelines for submitting code & software for further information.

Data

Policy information about availability of data

All manuscripts must include a data availability statement. This statement should provide the following information, where applicable:

- Accession codes, unique identifiers, or web links for publicly available datasets
- A description of any restrictions on data availability
- For clinical datasets or third party data, please ensure that the statement adheres to our policy

All minimum data to reproduce the figures are deposited into the synapse public repository (https://www.synapse.org/VirtualBiopsySystem). A sign-in process is required to access the data. Full source data are available from the corresponding author.

Research involving human participants, their data, or biological material

Policy information about studies with human participants or human data. See also policy information about sex, gender (identity/presentation), and sexual orientation and race, ethnicity and racism.

Reporting on sex and gender	In this research, we included donor sex variable. Both sex were included and they were self-reported and the number and percentage were reported in the manuscript. In the derivation cohorts, 5450 (44.0%) were female. There were 11 (0.1%) missing sex data in the derivation cohort. In the external validation cohort, 723 (44.4%) were female and no missing sex data.
Reporting on race, ethnicity, or other socially relevant groupings	In this research, we included black, white, and other ethnicity as well. Others includes Hispanic, Asian, and Arabic. In the derivation cohorts, 63 (0.7%) were black, 3329 (34.7%) were white, and 232 (2.4%) were others. There were 5967 (62.2%) missing ethnicity data in the derivation cohort. In the external validation cohort, 195 (11.9%) were black, 851 (51.8%) were white, and 596 (36.3%) were others. There was no missing ethnicity data in the external validation cohort. Although included, we did not include the ethnicity variable as an independent variable due to the limitation of data access in Europe.
Population characteristics	In the study, we included adult (over or equal to 18 years old) cohort. Living or deceased and transplanted or discarded adult donors for kidney transplantation were included. Day-zero biopsies were collected for the analysis. In the derivation cohort, the mean donor age was 46.7 ± 14.9 (SD) years and 9,395 (75.8%) were deceased donors. The mean serum creatinine was 1.2 ± 1.0 mg/dL. In the external validation cohort, the mean donor age was 48.0 ± 13.2 (SD) years and 1,124 (69.0%) were deceased donors. The mean serum creatinine was 1.6 ± 1.1 mg/dL. Detailed tables are available in the manuscript.
Recruitment	For the derivation cohort, the study involved 15 centers including 14 institutions from seven countries (France, Belgium, Croatia, Spain, United States, Canada, and Australia) and the largest Organ Procurement Organization (OPO) in the USA (OneLegacy). For the external validation cohorts, two institutions from two countries were involved: Columbia university medical center from the USA and Sun-Yat sen university form China. A total of 15,121 kidney biopsies were assessed overall.
Ethics oversight	All data were anonymized, and the clinical and biological data were collected from each center and entered into the Paris Transplant Group database (French data protection authority (CNIL) registration number 363505). On January 1st, 2021, the data were accessed from the database. On November 19th, 2021, the Chinese data were accessed from the database. On June 8th, 2022, the OneLegacy OPO data were accessed from the database. The protocol of this study (NCT04759209) was approved by the Paris Transplant Group's institutional review board. Written informed consent was given by all living donors at the time of transplantation. All data from the Paris Transplant Group centers (Necker, Saint Louis, and Toulouse Hospitals) were entered prospectively at the time of transplantation; a structured protocol was used to ensure harmonization across study centers. To ensure data accuracy, an annual audit was performed. As part of standard clinical procedures, other datasets from the European, North American, Australian, and Asian centers were compiled, entered in the databases of the centers in accordance with local and national regulatory standards, and submitted to the Paris Transplant Group anonymously.

Note that full information on the approval of the study protocol must also be provided in the manuscript.

Field-specific reporting

Please select the one below that is the best fit for your research. If you are not sure, read the appropriate sections before making your selection.

☒ Life sciences ☐ Behavioural & social sciences ☐ Ecological, evolutionary & environmental sciences

For a reference copy of the document with all sections, see nature.com/documents/nr-reporting-summary-flat.pdf

Life sciences study design

All studies must disclose on these points even when the disclosure is negative.

Sample size	As the aim of our study was to develop the Virtual Biopsy System using the donor parameters to predict day-zero kidney biopsy lesions, we included as much kidney transplant biopsies as possible. We collected 14,032 adult biopsies from 17 referral centers in Europe, North America, Australia, and Asia; among them, 12,402 were in the derivation cohort and 1,630 were in the external validation cohorts. This represents one of the largest populations of well-phenotyped kidney transplant recipients studied to date.

Data exclusions	We excluded inadequate biopsies according to Banff international classification requirements (n=1,089, 7.2%).
Replication	All minimal data, models, and codes to reproduce and to replicate the Figures are available in the synapse public repository (https://www.synapse.org/VirtualBiopsySystem). Two investigators (DY, GD) have independently and successfully reproduced and replicated the Figures, by using these data and codes.
Randomization	During the repeated cross-validation process, we used stratified random sampling technique to perform internal validation.
Blinding	Since the Virtual Biopsy System study is a development of machine learning-based models, blinding is not applicable to the study.

Reporting for specific materials, systems and methods

We require information from authors about some types of materials, experimental systems and methods used in many studies. Here, indicate whether each material, system or method listed is relevant to your study. If you are not sure if a list item applies to your research, read the appropriate section before selecting a response.

Materials & experimental systems

n/a	Involved in the study
☒	☐ Antibodies
☒	☐ Eukaryotic cell lines
☒	☐ Palaeontology and archaeology
☒	☐ Animals and other organisms
☐	☒ Clinical data
☒	☐ Dual use research of concern
☒	☐ Plants

Methods

n/a	Involved in the study
☒	☐ ChIP-seq
☒	☐ Flow cytometry
☒	☐ MRI-based neuroimaging

Clinical data

Policy information about clinical studies

All manuscripts should comply with the ICMJE guidelines for publication of clinical research and a completed CONSORT checklist must be included with all submissions.

Clinical trial registration	NCT04759209
Study protocol	Full trial protocol can be accessed at ClinicalTrials.gov: NCT04759209.
Data collection	The population consisted of living or deceased and transplanted or discarded adult donors for kidney transplantation enrolled from January 1st, 2000, to December 31st, 2021, who underwent kidney biopsies performed prior to kidney transplantation as part of standard of care. For the derivation cohort, the study involved 15 centers including 14 institutions from seven countries (Necker Hospital, Paris, France; Saint-Louis Hospital, Paris, France; Toulouse Hospital, Toulouse, France; Bicêtre Hospital, Kremlin Bicêtre, France; University Hospital, Leuven, Belgium; University Hospital, Liege, Belgium; University Hospital Centre Zagreb; Zagreb, Croatia; Hospital Clínic i Provincial de Barcelona, Barcelona, Spain; Hospital Vall d'Hebrón, Barcelona, Spain; Bellvitge University Hospital, Barcelona, Spain; Mayo Clinic, Rochester, MN, USA; University of British Columbia, Vancouver, BC, Canada; University of Alberta, Edmonton, AB, Canada; and Adelaide Hospital, Adelaide, Australia) and the largest Organ Procurement Organization (OPO) in the USA (OneLegacy). For the external validation cohorts, two institutions from two countries were involved: Columbia university medical center from the USA and Sun-Yat sen university from China. A total of 15,121 kidney biopsies were assessed overall. Exclusion criteria were inadequate biopsies according to Banff international classification requirements (n=1,089, 7.2%). A total of 14,032 kidney allograft biopsies were included for the final analyses including 1,372 (9.8%) from discarded kidneys. Among them, 12,402 were in the derivation cohort and 1,630 were in the external validation cohorts.
Outcomes	The primary outcomes were the four biopsy lesion findings by expert pathologists from each center according to the international Banff classification of allograft pathology, which uses a validated semi-quantitative ordinal grading scheme for all kidney compartments including: i) arteriosclerosis defined by arterial intimal thickening in the most severely affected artery (Banff "cv" score), ii) arteriolar hyalinosis defined by periodic acid-Schiff (PAS)-positive arteriolar hyaline thickening (Banff "ah" score), and iii) interstitial fibrosis and tubular atrophy (Banff "IFTA" score), computed with the extent of cortical fibrosis (Banff "ci" score) and cortical tubular atrophy (Banff "ct" score). Last, the continuous percentage of sclerotic glomeruli was defined by the percentage of the total number of glomeruli affected by global sclerosis ("glomerulosclerosis" score). No secondary outcome was included in this study.

Plants

Seed stocks	*Report on the source of all seed stocks or other plant material used. If applicable, state the seed stock centre and catalogue number. If plant specimens were collected from the field, describe the collection location, date and sampling procedures.*
Novel plant genotypes	*Describe the methods by which all novel plant genotypes were produced. This includes those generated by transgenic approaches, gene editing, chemical/radiation-based mutagenesis and hybridization. For transgenic lines, describe the transformation method, the number of independent lines analyzed and the generation upon which experiments were performed. For gene-edited lines, describe the editor used, the endogenous sequence targeted for editing, the targeting guide RNA sequence (if applicable) and how the editor was applied.*
Authentication	*Describe any authentication procedures for each seed stock used or novel genotype generated. Describe any experiments used to assess the effect of a mutation and, where applicable, how potential secondary effects (e.g. second site T-DNA insertions, mosiacism, off-target gene editing) were examined.*
